# Meiotic progression in multinuclear mouse spermatocytes without the spindle pole clustering motor protein KIFC1 or cytokinesis forms single-cell late-stage spermatids

**DOI:** 10.1038/s41598-025-20463-2

**Published:** 2025-10-17

**Authors:** Calvin Simerly, Carrie Hartnett, Ashley Zyhowski, Emily Robertson, Caleb Harrison, In Ki Cho, Charles Easley, Gerald Schatten

**Affiliations:** 1 Departments of Cell Biology, Ob-Gyn-Repro Sci,Bioengineering, and UPMC Hillman Cancer Center, Pittsburgh Development Center of Magee-Womens Research Institute, University of Pittsburgh Medical Center, 204 Craft Avenue, Pittsburgh, PA 15213 USA; 2 Environmental Health Science and Regenerative Bioscience Center, College of Public Health, Edgar L. Rhodes Center for Animal and Dairy Science, 425 River Rd, Athens, 30602 GA Greece; 3 Present Address: Department of Women’s and Reproductive Health, Level 3 Women’s Centre, University of Oxford, John Radcliffe Hospital, Headington, Oxford UK

**Keywords:** Polynuclear spermatocytes, Centrioles, Microtubules, KIFC1, TACC3, Meiosis, mitosis, cell cycle, reproduction, development, cytokinesis, Cell biology, Developmental biology

## Abstract

Mammalian spermatogenesis is a highly dynamic process involving male germ cell self-renewal and terminal differentiation into progenitor cells that produce testicular sperm in coordination with testicular somatic support cells. Multinuclear giant cells identified in mutant, toxin-exposed or genetically altered mice post-meiosis, reflect abnormal spermiogenesis. Here, analysis of fixed isolated testicular cells immunolabeled for microtubules, molecular motors, spindle binding proteins, and DNA from non-transgenic and GFP-CETN2-expressing CB6F1 or C57BL mice identify polynuclear primary spermatocytes within the normal testicular cell population. We hypothesize polynuclear primary spermatocytes arise from spermatogonia through aberrant cytokinesis or cell fusion of tethered spermatogonia. Polynuclear primary spermatocytes apparently progress in pre-prophase-I stages, completing first centriole duplication and homologous chromosome pairing/DNA cross-over events, forming multiple bipolar metaphase-I spindles of distinct phenotypes based on hypothesized spermatogonia derivation but without the microtubule clustering motor protein KIFC1. Remarkably, polynuclear spermatocytes are consistent with completing meiosis without undergoing first or second cytokinesis, seemingly generating polyploid spermatids post-meiosis. Polyploid spermatids perhaps undergo cytodifferentiation, forming sperm axonemes, acrosomes, centrosome nuclear binding, transient manchette microtubule assembly for nuclear shaping, associating with Sertoli cells and entering suspected spermiation. Collectively, these findings demonstrate remarkable testicular plasticity post-differentiation and the lax cell-cycle control of spermatogenesis, regardless of nuclear constituency.

**Supplementary Information:**

The online version contains supplementary material available at 10.1038/s41598-025-20463-2.

## Introduction

Mammalian spermatogenesis is a highly choreographed, complex process in males, essential to produce viable sperm for fertilization and species propagation^1,2^. Within the testis are the seminiferous tubules (ST) that house basement membrane spermatogonial stem cells (SSCs), which both self-renew to maintain a pool of SSCs and produce the differentiating cells that give rise to testicular sperm for final modification and shaping in the epididymis. Differentiating spermatogonia produced by SSC division sequentially produce cells in a lineage-specific pattern by mitotic progression in a series of amplified divisions—type A progenitor→differentiating type A→differentiating type B—which produce long chains of spermatogonia connected by intercellular bridges (ICBs) that prevent abscission after cell division. This connectivity provides genetic equivalency to spermatogonia by passing critical factors and/or organelles through the ICB^3^. These events all occur at the basement membrane of the ST. Ultimately, mitotic spermatogonia stop mitosis and form primary spermatocytes, which migrate across the testis-blood barrier and enter the ST abluminal region. Primary spermatocytes then initiate meiosis reductional divisions, with a single primary spermatocyte giving rise to four haploid round spermatids. Spermiogenesis continues as a series of cytodifferentiation events to complete testicular sperm formation, including assembly, from the centrosomes, of the sperm axonemes for motility, acrosome production on the anterior sperm head to store critical enzymes for future oocyte interaction and penetration, and assembly of a microtubule-based structure called the manchette to shape the sperm head into the species-specific final form. Spermiation is the last step in elongated spermatid remodeling prior to disengagement from the ST and release into the lumen for transport into the epididymis^4^. Within the ST, spermatogenesis is regulated closely by Sertoli cell interaction^5^. Outside of the ST, spermatogenesis is supported by the basement membrane peritubular myoid cells, which support structural ST integrity and secrete critical growth factors for spermatogenesis^6^, and interstitial Leydig cells for the critical testosterone production^4,7^.

Critical advances in understanding mammalian spermatogenesis have been derived from the study of mutant or gene knockout mice^8–11^, exposure to gonadotoxic therapies or environmental toxins^12–14^, and mammalian cryptorchid testis^15^. Multinuclear giant cells are commonly identified abnormal spermatocyte phenotypes in testicular sections after genetic manipulations or drug/toxin exposures, with many showing disruptions in normal meiotic progression, aberrant mitotic or meiotic cytokinesis, and nuclear abnormalities (vacuolization; fragmented DNA), along with primary spermatocyte apoptosis and general seminiferous tubal atrophy. Most identified multinuclear giant cells show very rare post-meiotic cytodifferentiation and these cells are linked to spermatid death or meiotic cytokinesis failure^16,17^.

Here, utilizing a transgenic animal modified to express centrin-2 in all centrioles^18^ (GFP-CETN2), along with non-transgenic CB6F1 and C57BL mice, we identify polyploid primary spermatocytes among the general testicular cell population released after the ‘squash’ technique of fixed mouse seminiferous tubules^19–22^. The advantage of these applications resides in the easy identification and superior imaging details permitted on whole intact polynuclear spermatocytes, unlike paraffin-embedded cut cryosections that restrict detailed spatial observations or disruptive chemical applications that destroy cellular architecture and protein antigenicity^22^. We initially identified primary polynuclear spermatocytes by nuclear numbers and general cellular diameters suggesting their derivation from specific errors in mitotic spermatogonia. Synaptonemal complex protein 3 (SYCP3)^23^ and direct imaging of GFP-CETN2-expressing centrioles showed polynuclear primary spermatocytes in leptonema pre-prophase-I substages that undergo first centriole duplication^19^. Cytoskeletal imaging showed assembly of metaphase-I bipolar spindles of unique phenotypes related to cellular diameter of polynuclear spermatocytes, perhaps reflective of their derivation from spermatogonia errors. Surprisingly, polyploid spindles lack KIFCI, a critical microtubule motor protein in cancer cells essential for the assembly of bipolar spindles in the presence of amplified centrosomes to avoid activating the cell death machinery^24^. Unlike cancer cells, polyploid metaphase-I spindles rarely show clustered bipolar spindles in the presence of amplified centrioles. Interestingly, polyploid telophase-I and -II spermatocytes undergo karyokinesis, forming multiple midbodies/ICBs, but fail both meiotic cytokinesis stages. We also identify polyploid round spermatids post-meiosis with nuclear numbers consistent with primary spermatocytes that progress successfully through meiosis. Remarkably, polynuclear round spermatids achieve many hallmarks of cytodifferentiation, associate with Sertoli cells during spermatid elongation and enter spermiation. Collectively, our findings suggest polynuclear primary spermatocytes are unique members of the normal testicular cell population, perhaps with the ability to produce testicular sperm of unknown viability.

## Results

We identified a variety of intact whole polyploid spermatocytes and spermatids released from para-formaldehyde fixed seminiferous tubules after applying the ‘squash’ technique^22^ or after isolating live cells from seminiferous tubules by a brief, gentle enzymatic exposure and fixing in absolute cold methanol (−20 °C)^20,25^. Both methodologies release a limited subset of the total testicular cellular population from seminiferous tubules with only a fraction of those isolated cells attaching to polylysine-coated coverslips for analysis, preventing accurate determination of the percentage of polynuclear spermatocytes and spermatids present relative to the total testicular cellular population. However, polynuclear spermatocytes are not rare in any given image sample preparation. From 43 Male mice, including 29 GFP-CETN2-expressing Males and 14 CB6F1 or C57BL non-transgenic Males utilized for this study, we identified 264 polynuclear spermatocytes or spermatids out of 1,194 cells analyzed (22%).

**Fig. 1:**
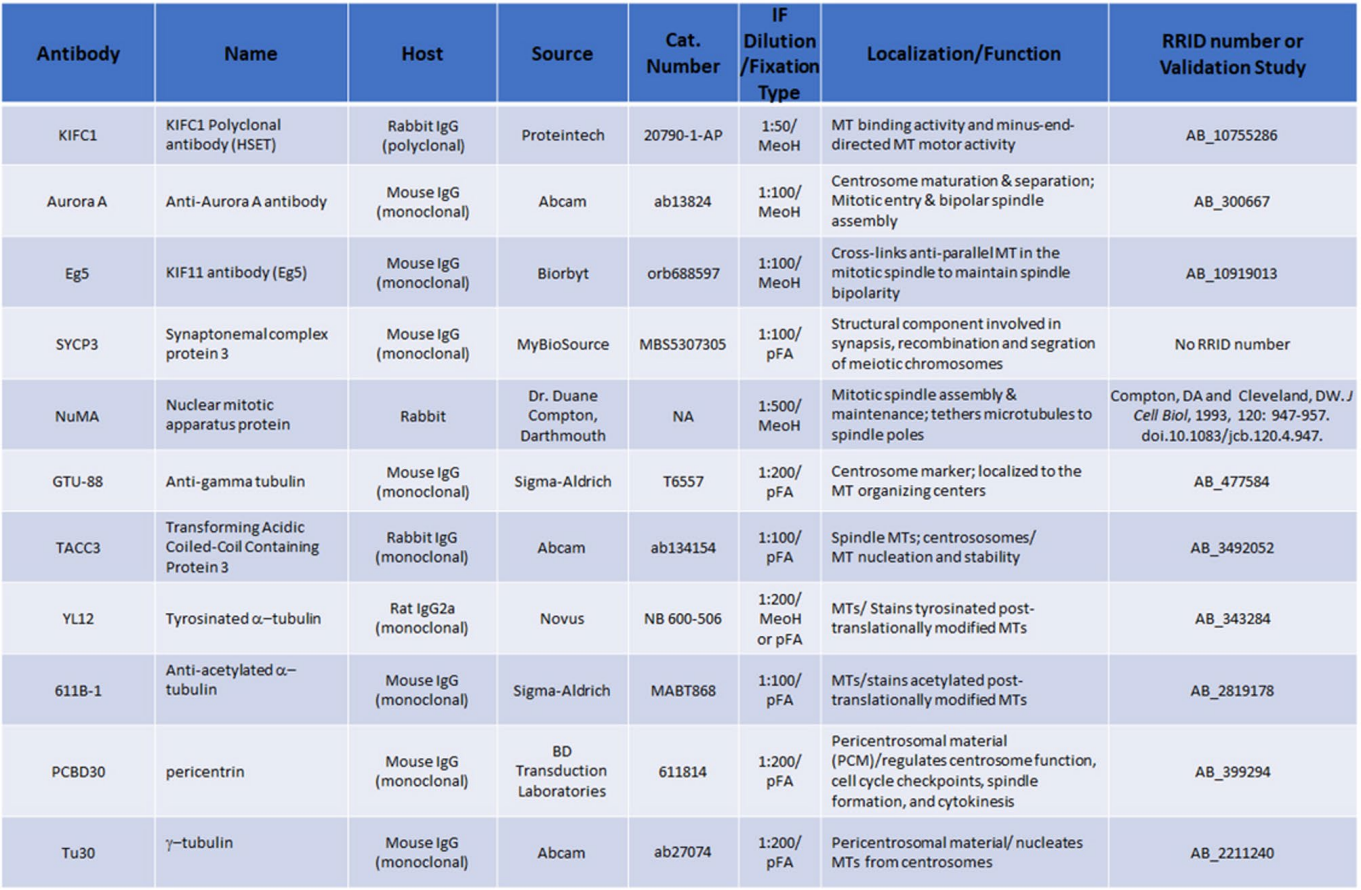
Primary antibodies utilized for investigating polynuclear spermatocytes and spermatids. The table includes sources, hosts, catalogue numbers, fixation type utilized, primary antibody dilutions, target functions and any Research Resource Identifier numbers (RRID) available for commercial antibodies or, alternatively, cited publications for antibody validation.

For investigating polyploid spermatocytes and spermatids, we utilized validated primary antibodies on fixed samples, including antibodies for microtubules (tyrosinated and acetylated α-tubulins)^26,27^; spindle molecular motor proteins KIFC1 [Kinesin-14], a minus-end microtubule spindle protein noted for its role in spindle pole clustering in cancer cells, among other functions^28^ and Eg5 [Kinesin-5], a microtubule crosslinking protein with plus-end directionality for spindle elongation, bipolarization, and clustering^29,30^; the microtubule spindle binding proteins NuMA (*Nu*clear *M*itotic *A*pparatus) involved in mitotic spindle pole focusing and positioning^31^ and Transforming Acidic Coiled-Coil 3 [TACC3]), a protein involved in spindle assembly and stabilization^20,32–34^; the phosphorylation regulatory kinase Aurora A, a serine/threonine protein kinase involved in spindle assembly and cell division^34–39^; and DNA stains (DAPI/Hoechst 33342) and Synaptonemal Complex Protein 3 (SYPC3), important for meiotic chromosome homologous pairing and DNA cross-over in pre-prophase-I meiotic spermatocytes^23^. Details of these probes, dilution and fixation techniques, and antibody validation characteristics are summarized in Fig. 1.

**Fig. 2:**
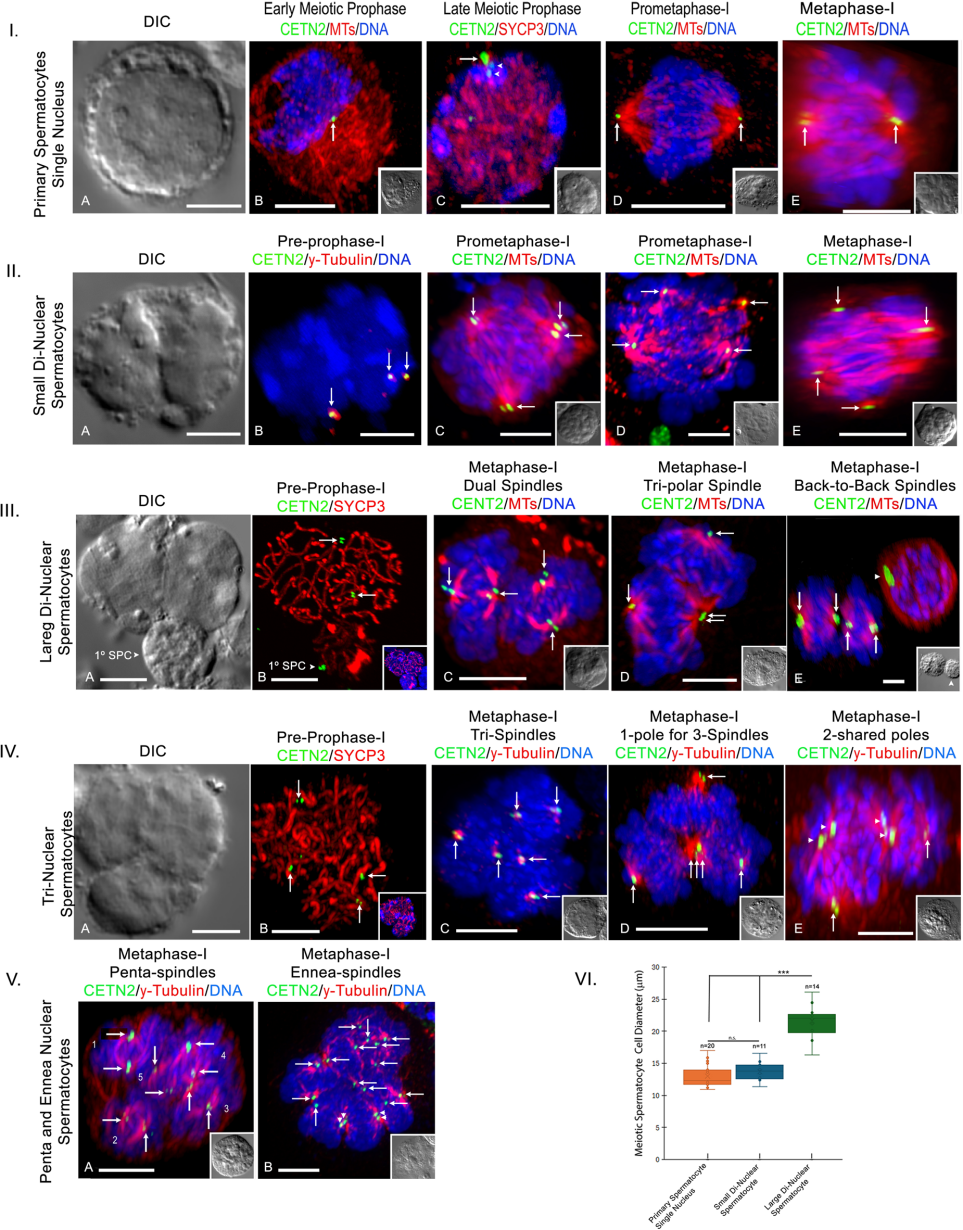
Polynuclear primary spermatocytes show unique first meiotic spindle phenotypes. **Panel I**: S*ingle nuclei primary spermatocytes* in differential interference contrast (**A**: DIC); pre-prophase-I (**B**) with unreplicated centrioles (green, arrow) and cytoplasmic microtubules (red); leptonema substage (**C**) with homologous chromosome pairing (red, SYCP3) and duplicated centrioles (green, arrowheads); prometaphase-I (**D**) and metaphase-I (**E**) bipolar spindles (red, microtubules) with centriole doublets (green, arrows) and aligning chromosomes (blue). **Panel II**: *Small di-nuclear primary spermatocyte* (**A;** DIC) at pre-prophase-I (**B**: blue, DNA) with replicated, split centriole doublets (green, arrows) within γ-tubulin (red); three meiosis-I spindles (**C-E**) show four assembling spindle poles (red, microtubules) demarked with centriole doublets (green, arrows) and central DNA alignment (blue). **Panel III**: *Large di-nuclear primary spermatocyte* (**A**; DIC; upper cell) with attached control leptonema spermatocyte (**A**: 1^◦^ SPC; arrowhead) at leptonema (**B**) showing homologous chromosome pairing (red, SYCP3) and duplicated centrioles (green, arrows); metaphase-I spindle phenotypes (**C-E**; red, microtubules) include dual spindles (**C**), tripolar spindle with single shared pole (**D;** double arrows) and back-to-back spindles (**E**; arrowhead, control interkinesis spermatocyte). All poles have centriole doublets (green, arrows) with chromosomes aligning on separate spindle equators (blue). **Panel IV**: *tri-nuclear primary spermatocytes* (**A;** DIC) at leptonema (**B**) with homologous chromosome pairing (red, SYCP3) and early centriole duplication (green, arrows); metaphase-I spindle phenotypes include tri-spindles (**C**: red, microtubules) each with aligning chromosomes (blue, DNA); 1 spindle pole for 3 spindles (**D**: red, microtubules; triple arrows) but separate aligning chromosomes (blue, DNA); and two shared spindle poles (**E**; red, microtubules; arrowheads) with separate chromosome alignment (blue, DNA). Poles have centriole doublets (**C-E**: green, arrows). **Pane V**: *penta (5) and ennea (9) polynuclear metaphase-I spindles* showing separate bipolar spindle assembly (red, microtubules), spindle pole centriole doublets (green, arrows), some with shared poles (B: arrowheads). Note individual aligning chromosome sets (blue, DNA.) **Panel VI.** Left: cell diameters in single nucleus (orange), small di-nuclear (purple) and large di-nuclear spermatocytes (green). Control and small di-nuclear spermatocytes have similar cell diameters (*p* < 0.2451) but differ significantly from large di-nuclear spermatocytes (***; *p* < 0.0001). All insets: DIC, except panels III.B and IV.B, DIC and SYCP3 (red). Scale bars = 5 μm.

Figure 2, panel I shows a classical isolated control single nucleus primary spermatocyte in DIC (cell diameter = 13 μm) and at pre-prophase-I with cortical microtubules and a single centriole doublet pre-replication (Fig. 2B; cell diameter = 12-µm). At leptonema substage, control primary spermatocytes immunostained with SYCP3 show paired homologous chromosomes with first centriole duplication (Fig. 2C; cell diameter = 12 μm)^19^. Meiotic prometaphase and metaphase spermatocytes assemble single bipolar meiotic spindles with a pair of centriole doublets at each pole (Figs. 2D and E, 13 and 14-µm, respectively), as previously shown^19,20,25^.

A typical isolated small di-nuclear primary spermatocyte in DIC is shown in Fig. 2, panel IIA (cell diameter = 12 μm). Like control spermatocytes, leptonema substage pre-prophase-I small di-nuclear primary spermatocytes undergo GFP-CETN2 centriole duplication and early splitting with centriolar γ-tubulin organization (Fig. 2, panel IIB, double arrows)^19^. But, unlike control spermatocytes, small di-nuclear spermatocytes assemble multipolar metaphase-I spindles with a pair of GFP-CETN2 doublets at each pole and dual chromosome sets intermixing centrally between the poles (Fig. 2C, D, and E; arrows; cell diameters = 14-µm, 13 μm, and 12-µm; 23/23; 100%). Overall, small bi-nuclear meiotic spermatocytes represented 16% of the polynuclear primary spermatocyte population collected in this study (29/183; *n* = 12 males).

By contrast, a large di-nuclear primary spermatocyte (cell diameter = 22 μm) is shown in Fig. 2, panel IIIA, adjacent to a typical single nucleus control primary spermatocyte (1^O^ SPC, arrowhead; cell diameter, 10-µm). Both pre-prophase-I cells were positive for SYCP3 homologous chromosome labeling (Fig. 2, panel III B, red) and GFP-CETN2-tagged centrioles pre-duplication (Fig. 2, panel III B, arrows and arrowhead). During metaphase-I, large di-nuclear spermatocytes assembled distinct phenotypes in first meiosis (Fig. 2, panel III C-E), including dual independent cytoplasmic spindles (cell diameter; 21-µm; 49/82; 60%), tripolar spindles with a single shared spindle pole (cell diameter, 19-µm; 30/82; 37%) and, rarely, back-to-back meiotic spindles (cell diameter = 16-µm; 3/82; 3%). In all cases, chromosomes did not intermix together as observed in small di-nuclear primary spermatocytes but aligned on separate spindle equators (Fig. 2, panel III, C-E; blue, DNA). Large bi-nuclear spermatocytes were the most numerous polynuclear meiotic spermatocytes observed (111/183; 61%; *n* = 23 males).

Tri-nuclear primary spermatocytes were also identified, here clearly showing three equal size nuclei (Fig. 2, panel IVA; DIC; cell diameter = 18-µm) with SYCP3 homologous chromosome labeling and asynchronous GFP-CETN2 expressing centriole duplication (Fig. 2, panel IV B; note four CETN2 doublets, arrows). As observed in large di-nuclear meiotic spermatocytes, unique spindles assembled in tri-nuclear spermatocytes, each with aligning chromosome sets, including independent tri-spindles (Fig. 2, panel IV C; 9/20; 45%); a polyhedron-like spindle with a single pole for all three spindles (Fig. 2, panel IV D; 5/20; 25%); and one or two shared spindle poles (Fig. 2, panel IV.E, 6/20; 30%). Overall, we observed 36/183 (20%; 12 males) tri-nuclear spermatocytes in our study.

We found other configurations of polynuclear spermatocytes, including cells with four (quad), five (penta) or nine (ennea) independent assembled first meiotic spindles (Fig. 2, panel V B), some showing shared spindle pole patterns, and all with independent chromosome alignment on individual spindle equators (Fig. 2, panel V.B, arrowheads). Many of these larger polynuclear spermatocytes were damaged from the coverslip mounting step, thus accurate cell diameters were not possible. Perhaps rarer, only 4/183 (2%) of imaged meiotic polynuclear spermatocytes showed greater than 3 nuclei in our study.

Finally, in Fig. 1, panel VI, we provide a graphic depiction of cell diameter sizes for control single nuclear primary spermatocytes, small di-nuclear and large di-nuclear polynuclear cells, demonstrating the similarity in size between control and small di-nuclear spermatocytes but significant difference with large di-nuclear spermatocytes. Taken together, and in conjunction with the unique spindle phenotypes observed in large di- or tri-nuclear primary spermatocytes, we hypothesize that small di-nuclear spermatocytes are derived after failed cytokinesis in mitotic spermatogonia while large diameter polynuclear cells are likely derived by cell-to-cell fusion of mitotic spermatogonia linked in syncytial-like chains.

Regardless of cell diameters, polynuclear first meiotic spermatocytes show rare spindle pole clustering ability in the presence of amplified centrosomes (Supple Fig. S1; 3/183; < 2%; *n* = 35 males). A classic example of spindle pole clustering is shown in a CF-1 somatic fibroblast cell line expressing multiple GFP-CENT2 centrioles in panel I. These fibroblasts strongly express the spindle pole clustering protein TACC3 where, perhaps through interaction with KFICI microtubule motor protein, assemble amplified centrioles into bipolar spindles to avoid activating the cell death machinery^24,40^. Meiotic polyploid spermatocytes do cluster amplified GFP-CETN2-expressing centrioles split between bipolar spindle poles, but with highly unaligned chromosomes (Supple S1, panels II and III). Interestingly, diffuse spindle TACC3 and its phosphorylating kinase Aurora A^41^, were found in clustered spindle poles of polyploid spermatocytes compared to tight centrosome patterns in control spermatocytes (Supple Fig. S1, panels II and II, images C and I)^20^. Thus, meiotic spindle pole clustering in polyploid primary spermatocytes with amplified centrosomes is uncommon, perhaps reflecting atypical patterns of TACC3 and Aurora A kinase spindle protein distribution.

**Fig. 3:**
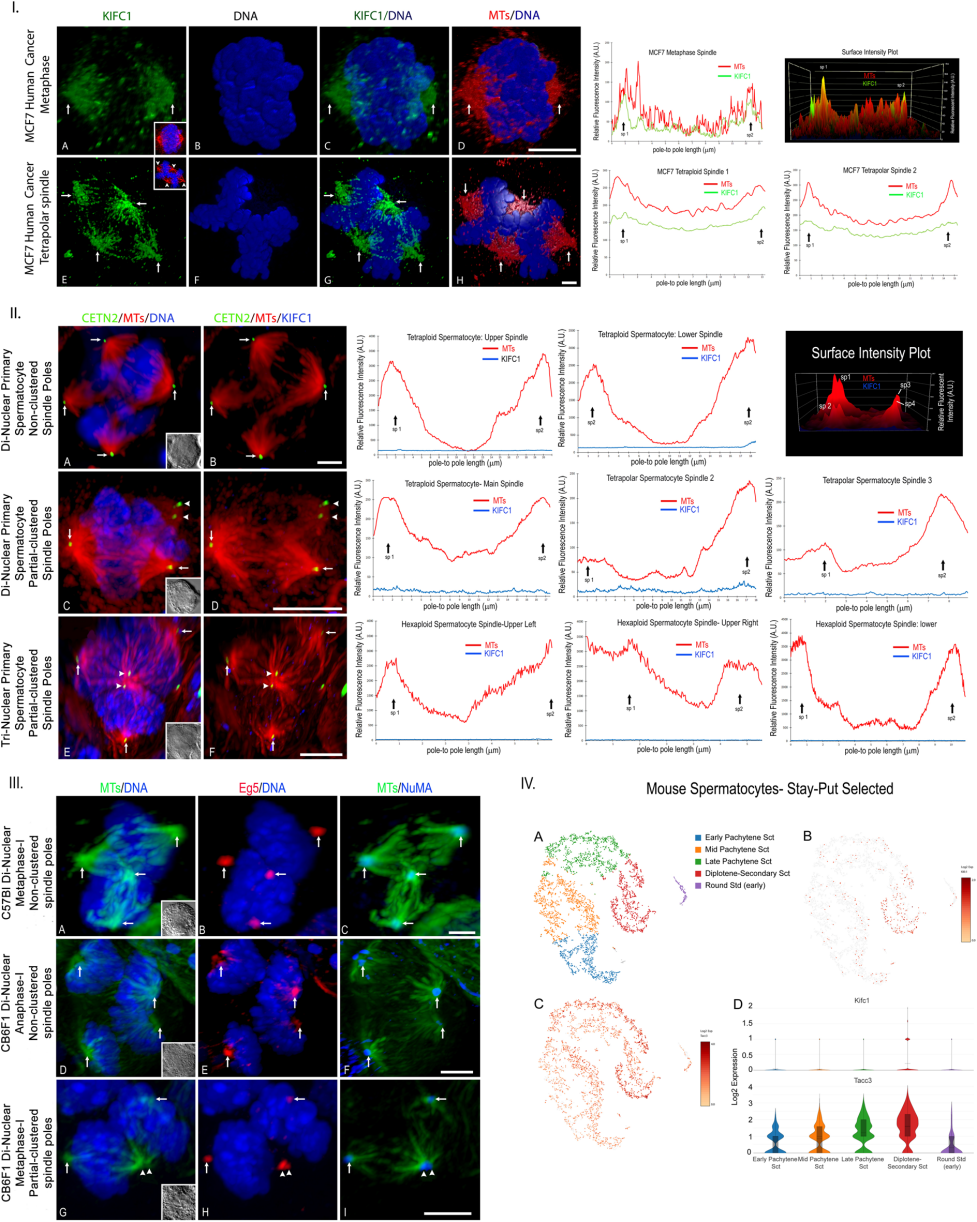
The microtubule motor clustering protein KIFC1 is not detected in polynuclear metaphase-I primary spermatocytes. *Panel 1.* Human MCF7 diploid (**A-D**) and polyploid (**E-H**) cancer cells showing robust spindle microtubule detection with KIFC1 (A, E: green, arrows, spindle poles; inset, microtubules [red] and DNA [blue]). B, F: blue, DNA; C and D: overlays. Volume rendering of microtubules (red), KIFC1 (green), and DNA (blue). Right: Relative fluorescent intensity line tracings and surface intensity plot (upper right) of the MCF7 control (upper graphs) or polyploid (lower graphs) spindles showing microtubules (red) and KIFC1 (green) spindle poles overlap (arrows, sp1, sp2). Scale bars = 5 μm. *Panel II.* GFP-CETN2-expressing polyploid meiosis-I spermatocytes do not detect KIFC1. (**A**,** C**,** E**) Centrioles (green, arrows), spindle microtubules (red) and DNA (blue) in di-nuclear (A, C) and tri-nuclear (E)-derived metaphase-I spindles. C, E: arrowheads, apposed spindle poles. **B**,** D**,** F**: Centrioles (green, arrows), spindle microtubules (red) but not KIFC1 (blue) detection of spindle microtubules (D, F: arrowheads, apposed poles). Right: fluorescent line intensity traces through the polyploid spindles show no KIFC1 detection (blue line) with spindle pole microtubules (red line; spindle poles, sp1 and sp2, arrows). Upper right: surface intensity plot confirming no KIFC1 detection in the di-nuclear meiotic spermatocyte four spindle poles (**A-B;** sp1→sp4). Scale bars = 5 μm. *Panel III.* The microtubule crosslinking motor protein KIF11 (Eg5; B, E, H: red, arrows) and the microtubule spindle pole binding protein NuMA (C, F, I: blue, arrows) are present in CB6F1 or C57BL mouse polyploid meiotic spindle pole microtubules. A, D, G: microtubules (green) and DNA (blue). Scale bars = 5 μm. *Panel IV.* Single cell RNA-seq analysis of KIFC1 and TACC3 mRNA expression in male adult Stay-Put enriched spermatocytes from Hermann et al.^47^. A: 10x Genomic tSNE plot profiling of selected adult mouse spermatogenic cells (key: color code). B and C: tSNE plots of KIFC1 (B) and TACC3 (C) expression on cell cluster showing minimal mRNA KIFC1 detection. Right: log2 expression color bar. D: Violin plots of KIFCI (upper) and TACC3 (lower) mRNA expression depicting limited KIFC1 distribution and density in various spermatocyte populations, compared to TACC3 mRNA.

Why meiotic polynuclear spermatocytes fail spindle pole clustering in the presence of amplified centrioles was further examined (Fig. 3). KIFC1 (kinesin 14) is a minus-end directed microtubule crosslinking motor protein vital for bipolar spindle assembly, centrosome integrity, chromosomal segregation, and spindle pole focusing, reportedly detected in mouse spermatocytes^42^. Here, a rabbit polyclonal KIFC1 antibody validated in Western blots of mammalian testis tissues (mice, human) and cell lines (Proteintech group; https://www.ptglab.com)^43^ was applied to methanol fixed human MCF7 cancer cells strongly labeling spindle microtubules, including in multipolar spindles (Fig. 3, panel 1, left). Mitotic spindle fluorescent intensity line tracings and surface plots measurements showed strong spindle pole KIFC1 detection along with spindle microtubules (Fig. 3, panel 1, right graphs). Conversely, meiotic polynuclear spermatocytes showed no KIFC1 spindle microtubule localization (Fig. 3, panel II, left), confirmed by fluorescent intensity measurements (Fig. 3, panel II, right graphs). Both Eg5 (kinesin 5) and the spindle associated protein NuMA, other known spindle pole clustering proteins, were detected in polynuclear spermatocyte meiotic spindles (Fig. 3, panel III)^44,45^. Analysis of a mouse spermatogenesis single cell RNA-seq Transcriptome database from 4,233 Stay-Put selected adult mouse spermatocytes^46,47^ showed significant TACC3, but not KIFC1, mRNA detection in diplotene- secondary selected spermatocytes (Fig. 3, panel IV), supporting the lack of KIFCI spindle staining in meiotic polynuclear spermatocytes. Collectively, polynuclear spermatocytes lacking spindle pole KIFC1 cannot cluster amplified centrosomes into a single bipolar spindle and the presence of other known clustering protein like Eg5 and NuMA do not compensate for KIFC1 spindle pole absence.

**Fig. 4:**
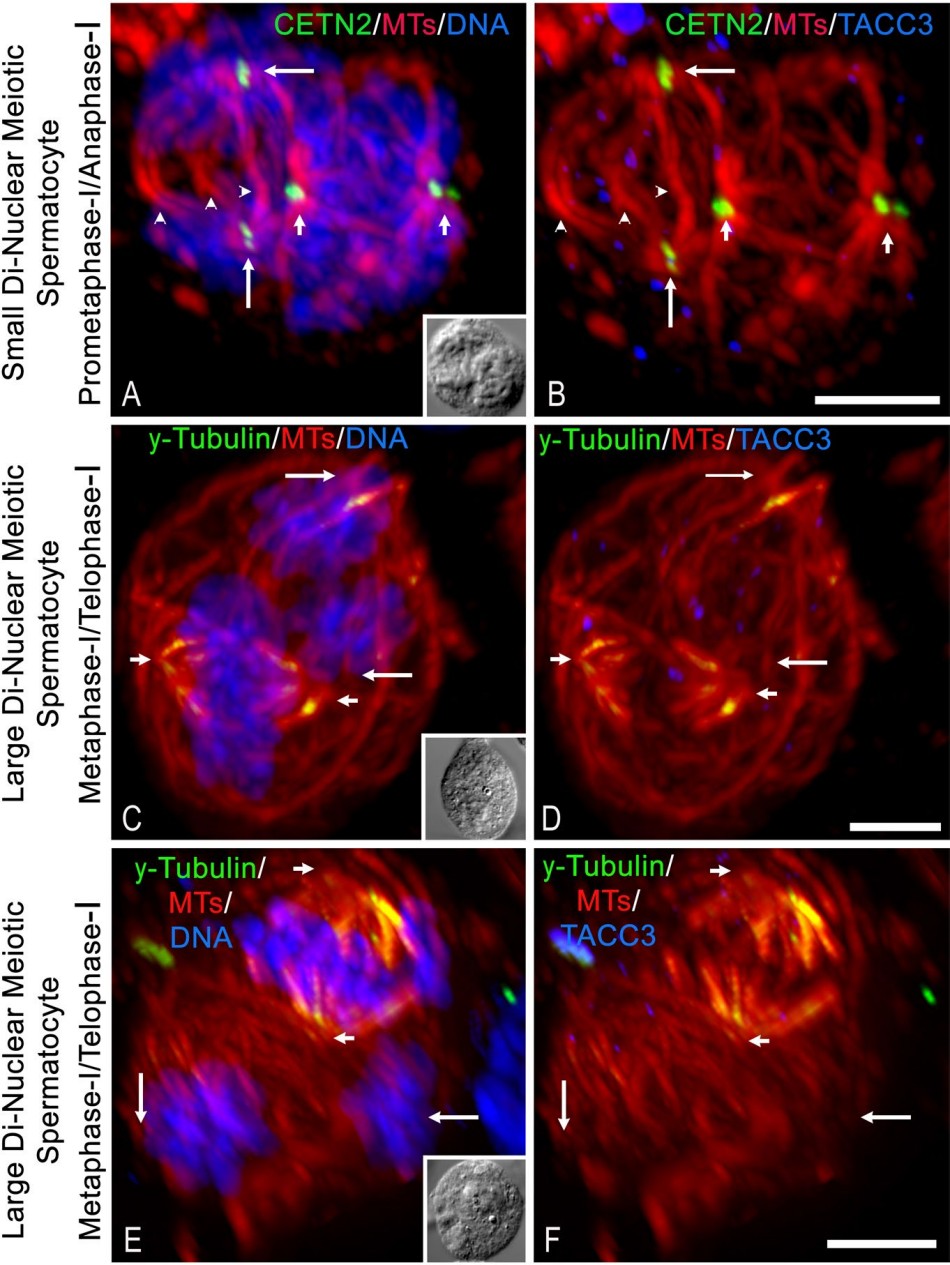
Fusion-derived polyploid primary spermatocytes can show asynchronous progression of meiotic spindles within a common cytoplasm. (**A-B**) GFP-CETN2-expressing small di-nuclear meiotic spermatocyte with two independent bipolar spindles (red, microtubules): prometaphase-I (right; short arrows, duplicated spindle pole centrioles) with aligning chromosomes (blue) and late anaphase-I (left; long arrows, duplicated spindle pole centrioles) with properly segregated chromosomes at the spindle poles (blue) and an early developing midbody (arrowheads). Inset, DIC. (**B)** spindle pole TACC3 (blue) is not robustly observed in either the prometaphase (right; red, microtubules; green, centrioles, short arrows) or late anaphase spindles (left; red, microtubules; green, centrioles, long arrows). Estimated cell diameter = 15 µM; no cell cleavage initiation. **(C-F)** two non-GFP CETN2 large di-nuclear meiotic spermatocytes with asynchronous progressing spindles immunolabeled for GTU-88 γ-tubulin (green), spindle microtubules (red) and either DNA (C, E: blue) or TACC3 (D, F: blue). Metaphase-I spindles (short arrows demark bipolar spindle poles; red, microtubules) with aligned chromosomes at the spindle equator (blue), GTU88 γ-tubulin within the spindle microtubules (green) but no TACC3 observed at the spindle poles (D, F: blue). Telophase-I spindles (long arrows demarking spindle poles adjacent to separated chromosomes, blue) with a poorly assembled midbody (red, microtubules), weak spindle GTU88 γ-tubulin (green), and no spindle pole TACC3 (D, F: blue). C, E: insets: DIC, estimated cell diameters are 17 μm and 20 μm, respectively; no cell cleavage initiation. All scale bars = 5 μm.

Within the population of large di-and tri-nuclear meiotic polyploid primary spermatocytes (*n* = 147; *n* = 5 males), about 8% (12/147) showed asynchronous progression of assembled spindles, including prometaphase-I and early telophase-I within the same cytoplasm (Fig. 4A-B) and metaphase-I and telophase-I mixed spindle development (Fig. 4C-F). Notably, bipolar metaphase spindles with aligned chromosomes assembled and telophase-I cells had properly segregated chromosomes but poorly formed midbodies, with no evidence of cleavage furrow assembly for cell division (Fig. 4, insets, DIC). These di-and tri-nuclear meiotic spermatocytes were probably fusion-derived, based on measured cell diameters and cytoplasmic spindle organization phenotypes characteristic of these polyploid spermatocytes.

**Fig. 5:**
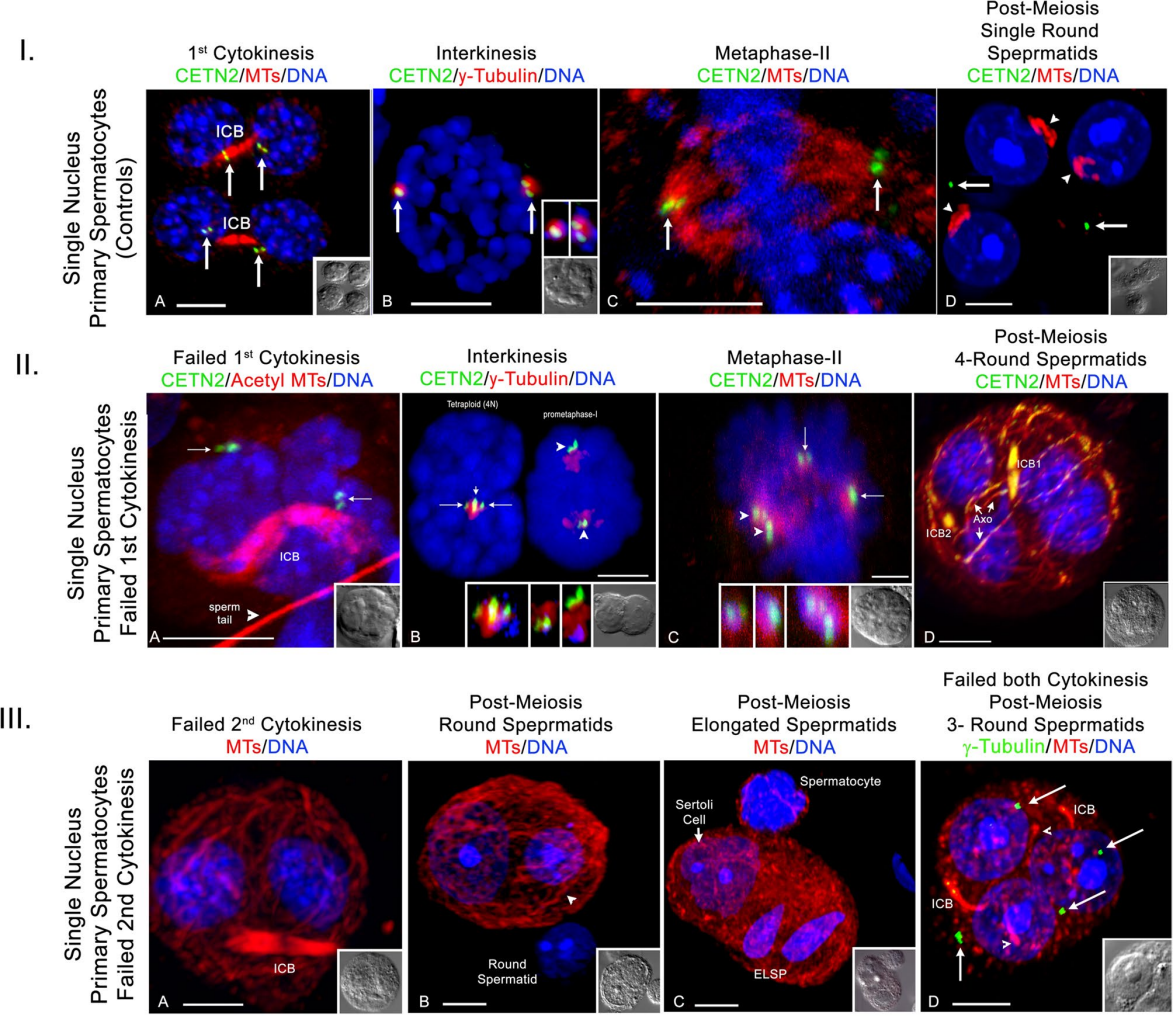
Meiotic control spermatocytes that fail first or second meiosis. **Panel I**. *Control primary spermatocytes.* (**A)** two post-first cytokinesis spermatocytes, each with one ICB (red, acetylated α-tubulin), two associated centriole doublets (green, arrows) and interkinesis DNA (blue). (**B**) interkinesis spermatocyte (blue, DNA) with fully duplicated and positioned centriole doublets (green; arrows) in pericentrin PCM (red; upper insets, details). (**C**) a bipolar metaphase-II spindle (red, microtubules) with centriole doublets (green, arrows) and aligning chromosomes (blue). (**D**) three post-meiotic round spermatids (RS) with acrosomal cap microtubules (red, arrowheads) and DNA with nucleoli (blue), but no centrioles expressing GFP-CETN2 (green). Arrows are centrioles outside RS cells. Insets: DIC. **Panel II.** *Failed first cytokinesis in control primary spermatocytes.* (**A**) telophase-I spermatocyte showing a single ICB (red, acetylated α-tubulin), separated chromosome sets (blue) and duplicated centrioles (green, arrows) without cleavage. (**B**) Left: non-cleaved di-nuclear spermatocyte (blue, DNA) with apposed centrioles (green, arrows; short arrow; adherent centrioles) within γ-tubulin. Right: prometaphase-I control spermatocyte with centrioles (green, arrowheads) within γ-tubulin PCM (red; middle insets, details) and aligning DNA (blue). (**C**) dual bipolar metaphase-II spindles (red, microtubules), each aligning separate chromosomes (blue). Spindle pole centriole doublets (green, arrows; left, middle insets, details) are observed, two closely apposed (green, arrowheads; inset, details). (**D**) a di-nuclear spermatocyte after failed second cytokinesis. Note early spermatid with two ICBs (green, γ-tubulin), 4-spermatid nuclei (blue) and nascent sperm axonemes (red, microtubules, axo). Insets, DIC. **Panel III**. *Failed second cytokinesis in control secondary spermatocytes.* (**A)** bi-nuclear (blue, DNA) early spermatid with single ICB (red, microtubules) but no cytokinesis (inset: DIC). (**B)** bi-nuclear RS with nucleoli (blue, DNA) and sperm axoneme assembly (red, microtubules; arrowhead). (**C)** bi-nuclear spermatid with two elongating spermatids (blue, DNA; ELSP) and a Sertoli cell (short arrow). (**D)** tri-nuclear spermatid with nucleoli (blue, DNA) showing two ICB’s and sperm axonemal assembly (red, microtubules; arrowheads). Note four γ-tubulin foci (green, arrows) associated with 3 nuclei, perhaps arising from both first second cytokinesis failure and second cytokinesis/karyokinesis errors. Insets: DIC. All scale bars = 5 μm.

Classical control single nucleus primary spermatocytes typically undergo cytokinesis at the end of first meiosis, forming two daughter cells connected by an ICB in close association with centriole doublets and with nuclei transitioning into interkinesis with unique heterochromatin sites (Fig. 5, panel IA; cell diameters, both 10-µm)^19^. During interkinesis, control primary spermatocytes initiate second centriole duplication in preparation for assembling metaphase-II bipolar spindles (Fig. 5, panel IB and C; cell diameters, both 12-µm)^19^. After completion of second meiosis, early round spermatids with a single nucleus, demarked by prominent nucleoli, and a centriole pair are observed (Fig. 5, panel ID; cell diameters = 12-µm).

We observed a small, rare population (33/933; 4%; *n* = 35 males) of control primary spermatocytes that failed first and/or second cytokinesis after meiotic telophase chromosome separation, forming polynuclear spermatocytes or spermatids (Fig. 5, panels II and III). Control spermatocytes that fail first cytokinesis show dual interkinesis nuclei, a single midbody/ICB, and two centriole doublets, some juxtaposed at the interface between separated nuclei, perhaps just prior to centriole duplication onset (Fig. 5, panel IIA-B; insets, DIC; cell diameter = 13-µm and 12- µm; 19/33; 58%). We hypothesize that these control di-nuclear cytokinesis failures may continue meiotic development, forming dual metaphase-II spindles with four poles, each having centriole doublets and aligning chromosomes along a central metaphase equator (Fig. 5, panel IIC; inset DIC; cell diameter = 16-µm; *n* = 2). Post-meiosis, we observed single round spermatids with 4-nuclei, two cytoplasmic midbodies/ICBs, and early sperm axoneme assembly, perhaps reflecting a di-nuclear spermatid derived from failure of first and second cytokinesis (Fig. 5, panel IID; inset, DIC; cell diameter = 23-µm; 8/33; 24%).

Interestingly, rare di-nuclear spermatids have also been found in our sample preparations and we hypothesize these cells are derived after failed second meiotic cytokinesis to produce early spermatids with two nuclei separated by a large ICB, visible sperm axonemal assembly and prominent nucleoli (Fig. 5, panel IIIA-B; insets, DIC; cell diameters = 12-µm; 6/33; 18%). We also identified later stage spermatids with two elongating nuclei, a Sertoli cell nucleus and abundant cytoplasmic microtubules (Fig. 5, panel IIIC; inset, DIC; cell diameter = 17-µm; *n* = 2). Variations included tri-nuclear spermatids with nucleoli, four γ-tubulin centrosomes, two-ICBs and assembled sperm axonemes, perhaps consistent with derivation from failed cytokinesis and karyokinesis at the end of meiosis (Fig. 5, panel IIIC, D; cell diameter = 18-µm; *n* = 2). Taken together, polynuclear spermatids from control spermatocytes are perhaps derived from failed first and/or second cytokinesis and are consistent with the capability of progressing in spermiogenesis post-meiosis.

**Fig. 6:**
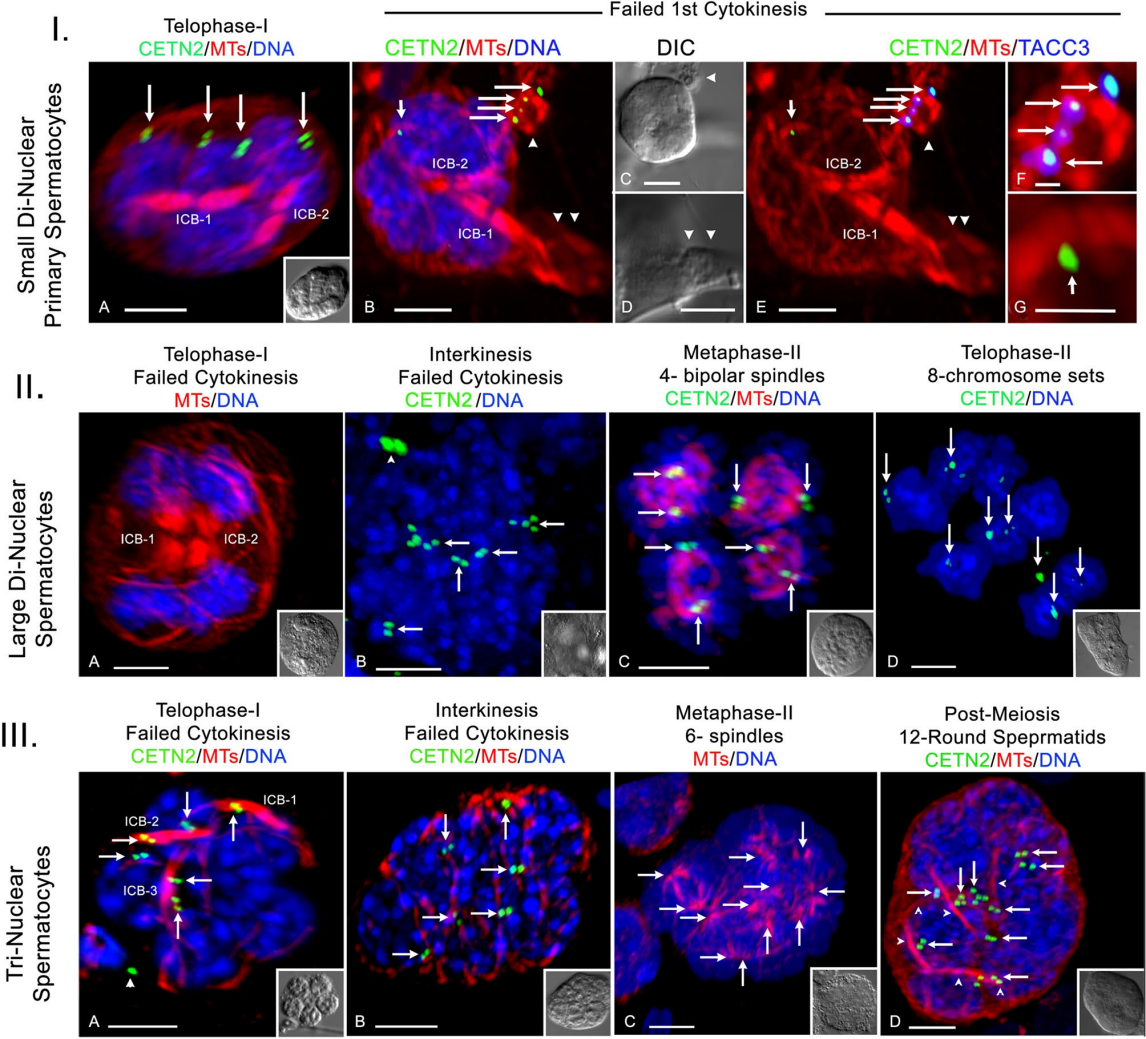
Polynuclear GFP-CETN2-expressing primary spermatocytes fail meiotic cytokinesis, perhaps forming polynuclear spermatids. **Panel I**. *Small di-nuclear primary spermatocytes*. (**A**) telophase-I di-nuclear spermatocyte showing 4- nuclei (blue), 2 ICBs (red, microtubules), and 4 centriole pairs (green, arrows) without cleavage (inset, DIC). (**B-G**) small di-nuclear telophase-I failure (C, D: DIC; arrowheads, cytoplasmic extensions) produced 4-nuclei (B: blue), two ICBs (B: red, microtubules) and misplaced centrioles (B: green, long arrows) with centriolar TACC3 (E: blue; F: details). The single retained cytoplasmic centriole (B: green, short arrow) is without TACC3 (E: blue; G: details). Both small di-nuclear telophase-I failures measured 11 μm. **Panel II**. *Large di-nuclear primary spermatocytes.* (**A)** telophase-I di-nuclear spermatocyte with two ICBs (red, microtubules) and 4 separate chromosome sets (blue, DNA) that failed first cytokinesis (inset, DIC; cell diameter, 22 μm). (**B)** interkinesis stage 4- nuclear single spermatocyte (inset, DIC; 20 μm) with 8 -centriole doublets (green, arrows; non-cell centrioles, arrowhead), indicating second centriole replication. (**C)** a metaphase-II spermatocyte with 4- independent spindles, each with aligning chromosome sets and spindle pole CETN2 doublets (green, arrows). Inset, DIC; cell diameter = 17 μm. (**D)** a telophase-II spermatocyte with 8-nuclei and GFP-CETN2 centrioles (green, arrows). Inset, DIC; cell diameter = 28 μm. **Panel III**. *Tri-nuclear spermatocytes.* (**A)** failed first cytokinesis telophase-I spermatocyte with 6- nuclei (blue, DNA), 3 ICB’s and 6 centriole doublets (green, arrows; arrowhead, testicular sperm centrioles). Inset, DIC; cell diameter 17 μm. (**B)** failed first cleavage spermatocyte shows 6 interkinesis nuclei (blue, DNA) with 6 centriole doublets (green, arrows) pre-second centriole duplication. Microtubules are cortical (red). Inset, DIC; cell diameter, 20 μm. (**C)** early metaphase-II spermatocyte with 6 assembling bipolar spindles (red, microtubules; arrows depict 12 spindle poles) around 6 condensing DNA sets (blue). Inset, DIC; cell diameter, 19 μm). (**D)** post-meiosis round spermatid with 12- nuclei (blue, DNA), 12 centriole pairs (green, arrows) and six ICBs (red, microtubules, arrowheads). Inset, DIC; cell diameter = 20 μm. Scale bars = 5 μm, except panel IF, G: 1 μm.

Polynuclear primary spermatocytes in later stages of meiosis were identified in our samples (46/264; 17%; *n* = 43 males). Small bi-nuclear primary spermatocytes appear to fail first cytokinesis, producing spermatocytes with two ICB’s without cleavage, four nuclei and 4-GFP CETN2 expressing cytoplasmic centrioles (Fig. 6, panel IA; *n* = 3; cell diameter = 10-µm; *n* = 4). Additionally, a single rare cell with aberrant first cytokinesis resulting in small cytoplasmic extensions with mis-placed centrioles, but no DNA content was observed (Fig. 6, panel IB-G; cell diameter = 11-µm; *n* = 1). Interestingly, small bi-nuclear primary spermatocytes beyond first meiosis did not appear in our samples.

Large bi-nuclear primary spermatocytes also failed first cytokinesis, demonstrating 4-nuclei and 2-ICBs but without cleavage (Fig. 6, panel IIA; *n* = 6; cell diameter = 22-µm). Post telophase-I, large bi-nuclear spermatocytes showed 4 interkinesis nuclei with 8-GFP-CETN2 expressing centriole doublets, suggesting accurate second centriole duplication (Fig. 6, panel IIB; *n* = 3; cell diameter = 20-µm). A large di-nuclear metaphase-II spermatocyte assembled four independent bipolar spindles, each spindle pole with GFP-CETN2 centrioles and separately aligning chromosomes on spindle equators (Fig. 6, panel IIC; *n* = 3; cell diameter 17-µm). A single spermatocyte at late telophase-II was also found with 8 segregated chromosome sets and 8-GFP-CETN2-expressing centriole doublets (Fig. 6, panel IID; cell diameter = 28-µm).

**Fig. 7:**
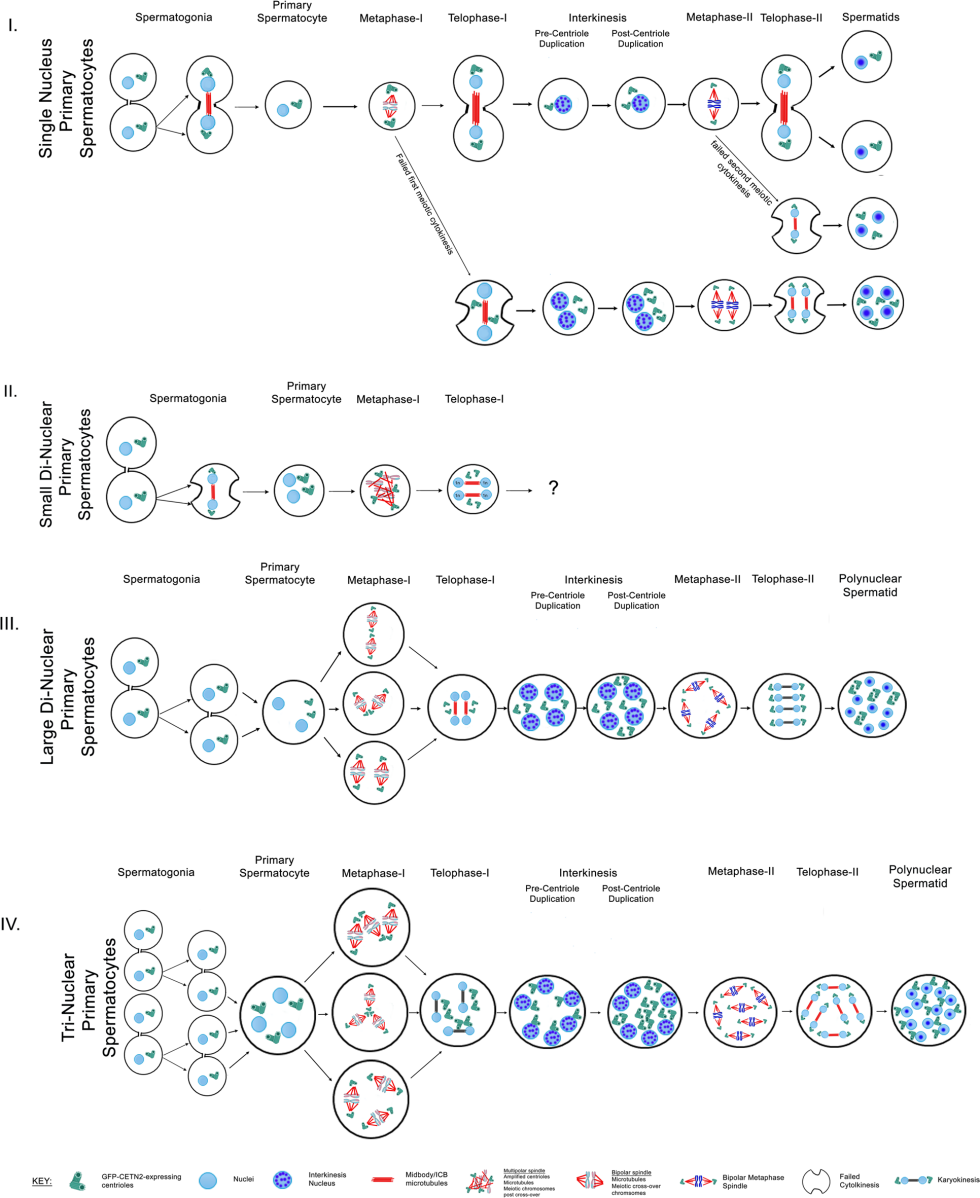
Potential pathways for mouse polynuclear spermatocyte generation and meiotic progression to polynuclear spermatids. Within the tightly choreographed mouse testis, polynuclear spermatocytes are found to be a viable subpopulation of mouse testicular cells able to achieve a variety of meiotic and post-meiotic stages. Measured parameters for classifying their attained developmental stage include general cellular diameters; number and chromosomal configuration of nuclei stained with Hoechst/DAPI dye; centriole doublet counts given their known first and second replication at leptonema substage of pre-prophase-I and interkinesis^19^; meiotic metaphase spindle assembly and chromosomal alignment; telophase midbody/ICB numbers with or without cleavage; and identity of prominent nuclear nucleoli, sperm axonemal assembly and acrosomal caps in early stage spermatids. Classical single nuclear primary spermatocytes generated from differentiating spermatogonia appear to produce polynuclear spermatocytes after failure of first and/or second cytokinesis, generating post-meiotic spermatids having four (first and second cytokinesis failure) or two (second cytokinesis failure) round spermatid nuclei. Centriole inheritance in these polynuclear spermatids are projected from metaphase-II bi-spindle configurations since GFP-CETN2 expression is silenced in early spermatids^25^ (**Panel I)**. Polynuclear primary spermatocytes prior to first meiosis are also common testicular cells. Small di-nuclear primary spermatocytes (**Panel II)** are perhaps derived from spermatogonia cytokinesis failure, being similar in size to classical primary spermatocytes (both ~ 13.01-µm). These di-nuclear spermatocytes initiate homologous chromosome pairing and DNA cross-over, along with first centriole duplication, forming metaphase-I spindles with four independent spindle poles and centrally aligned chromosomes. Rarely do di-nuclear spermatocytes cluster spindle poles, perhaps related to the absence of the molecular motor protein KIFCI. Small binuclear spermatocytes fail first cytokinesis and progression beyond first meiosis is not clear. Conversely, large diameter (> 20-µm) di- and tri-nuclear primary spermatocytes are found that also initiate homologous chromosomal pairing/DNA cross-over with faithful first centriole duplication (**Panels III and IV**). However, unlike small bi-nuclear spermatocytes, multiple spindles assemble at metaphase-I that align chromosomes on separate spindle equators. These large primary spermatocytes form multiple ICBs after successful first and second karyokinesis but fail cytokinesis, forming polynuclear spermatids with predicted round spermatid nuclei and centriole numbers. Bottom: schematic key.

We also found evidence of various meiotic developmental stages in tri-nuclear spermatocytes, including telophase-I with 6-nuclei, 3-ICBs and 6-GFP-CETN2-expressing centrioles without cell cleavage (Fig. 6, panel IIIA; *n* = 3; cell diameter = 17-µm), interkinesis stage having 6-nuclei and 6-GFP-CETN2-expressing centrioles pre-second centriole duplication (Fig. 6, panel IIIC; *n* = 3; cell diameter = 19-µm), and early second metaphase with 6-bipolar spindles aligning 6 individual chromosome sets (Fig. 6, panel IIIC; *n* = 2; cell diameter = 19-µm). Post-meiosis, early round spermatids with 12 nuclei, 12-GFP-CETN2-expressing centrioles and six ICBs were observed (Fig. 6, panel IIID; *n* = 3; cell diameter = 20-µm). Collectively, polynuclear spermatids of predictable nuclear and centriolar constitution are consistent with large polynuclear primary spermatocytes meiotic progression that fail first and second meiotic cytokinesis. These polynuclear spermatids appear viable as they assemble multiple sperm axonemes in parallel with nuclear numbers inherited. We provide a summary on polynuclear spermatocyte derivations and possible developmental pathways for producing polynuclear spermatids post-meiosis in Fig. 7 and Supplemental Table 1.

**Fig. 8:**
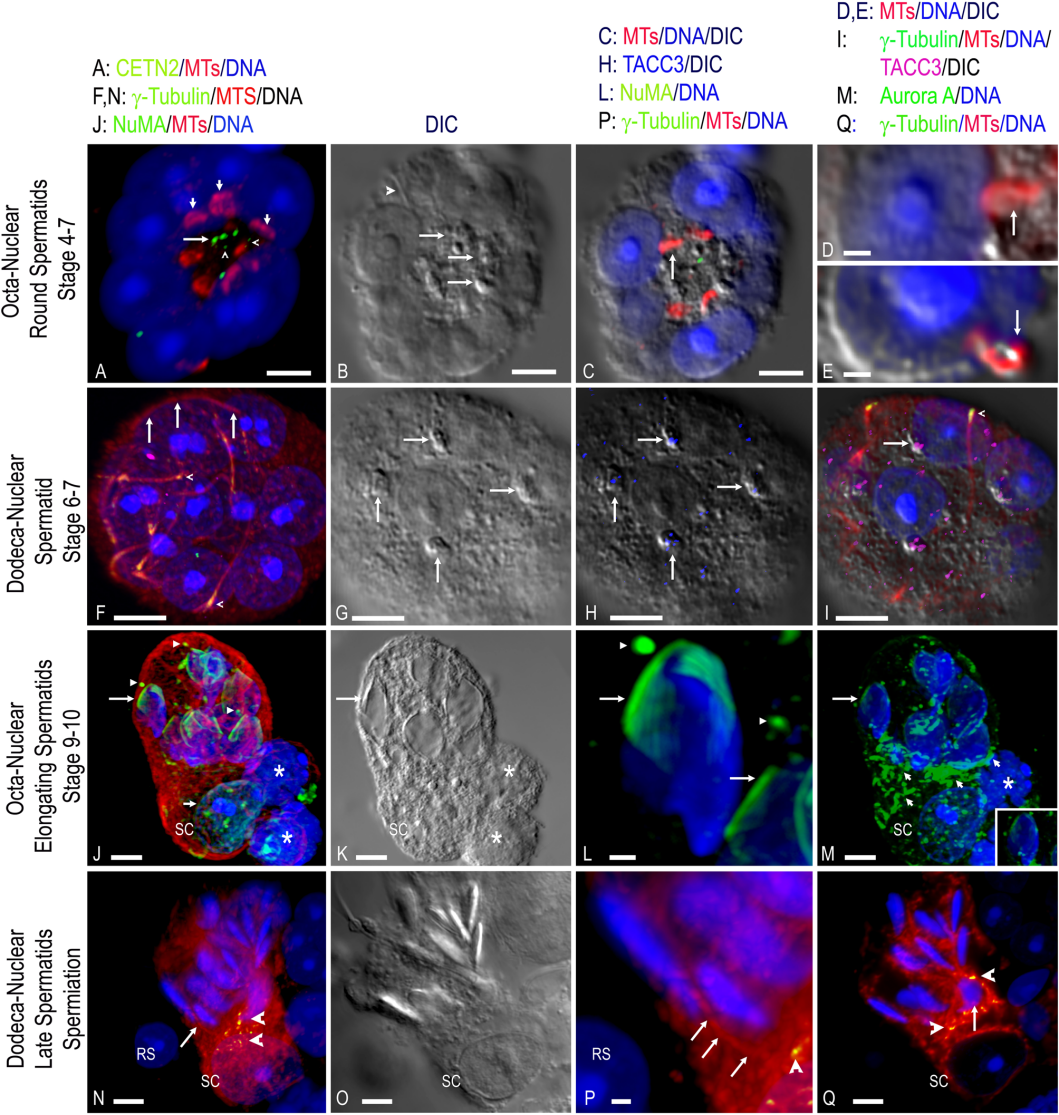
Polynuclear spermatids in spermiogenesis post-meiosis. **(A-E**) step 4-7octoploid round spermatid. (**A**) Octo-nuclear spermatid (blue, DNA) with only 4-GFP-CETN2-expressing centriole doublets (green, arrows; silencing GFP-CETN2, arrowheads) and microtubules at acrosomes (red, short arrows on 3 of 8 acrosomes; (**D**,** E**) details, triple overlay of acrosomal microtubules (red, arrows), DNA nucleus (blue), and DIC. (**B**) DIC; Golgi (arrows). (**C**) Triple overlay: DIC, microtubules (red, arrow) and DNA (blue). (**F-I**) step 6–7 dodecaploid round spermatid with sperm axonemes (red, microtubules), proximal end axonemal γ-tubulin (green, arrowheads) and nuclei with prominent nucleoli (blue, DNA). (**G**) cytoplasmic Golgi bodies (DIC; arrows). (**H**) overlay of DIC with TACC3 (blue) showing labeling at cytoplasmic Golgi (arrows). (**I**) quad overlay of DIC, DNA (blue), TACC3 Golgi labeling (magenta; arrow) and γ-tubulin PCM (green; arrowhead), showing tight centrosome: nuclear association on basal side of the nucleus opposite the Golgi/acrosomal cap. (**J-M**) multinuclear elongating spermatids with associated Sertoli cell (SC). (**J-L**) polarized elongating spermatids (blue, DNA) with assembled manchette microtubules (green, NuMA, arrow; L: details), spermatid centrosome labeling (green, NuMA, arrowheads; L: details) and Sertoli cell (short arrow; DNA, blue) with intranuclear NuMA (green). Cortical microtubules appear contiguous with the incorporated Sertoli cell (red, microtubules; K: DIC). *: two attached round spermatids. (**M**) aurora A kinase labels microtubules assembled in the Sertoli cell apical cytoplasm (green, short arrows) and weakly in manchette microtubules (green, long arrow; inset, details). (**N-Q**) a multinuclear spermatid cell in spermiation. 12 polarized late-stage spermatids (O: DIC) in a common cytoplasm with an attached Sertoli cell (SC). A SC-generated microtubule (red, arrow) interactions with one spermatid nucleus (DNA, blue; P: details). γ-Tubulin (green, arrowheads) is mainly in the SC apical cytoplasmic between the SC nucleus and the elongated spermatids. RS: round spermatid. An immature spermatid (Q: arrow; blue, DNA) with γ-tubulin (Q: green, arrowheads) at the elongated spermatid base has extensive microtubules (Q: red) enveloping the nucleus, some generated from the Sertoli cell (SC). All scale bars = 5 μm, except D, E, L, P: 1 μm.

We explored post-meiosis polynuclear spermatid development further (35/261; 13%; *n* = 9 males; Fig. 8 and Supple Figures S2-S5.) Early octa-nuclear spermatids showed microtubules bound on acrosomal caps (Fig. 8, A-E; Suppl Fig. 2S; 4/35; 11%; 3 males), but silenced GFP-CETN2 expression^25^, like found in control round spermatids (Supplemental Fig. S3 A-B).

Dodeca-nuclear (12; 6/35; 17%; *n* = 3 males) round spermatids at estimated step 6–7 showed advanced sperm axoneme assembly and centriole nuclear binding (Fig. 8, F-I; Suppl Fig. 2S). PCM γ-tubulin antibodies showed tight centrosomal nuclear localization, often opposite the Golgi/acrosome caps (Fig. 8F-I)^48,49^. TACC3 (Fig. 8, H-I; Supplemental Fig. S2 F, I, L, O) and KIFCI (Supplemental Fig. S4F) localized in Golgi/acrosomal caps, perhaps contributing to acrosome biogenesis^28,50^.

In later spermiogenesis steps, an octa-nuclear spermatid showed polarized elongating (ELSP) and round spermatid (RS) nuclei with an incorporated Sertoli cell (SC; Fig. 8K, DIC; Suppl Fig. 4; 3/35; 9%; 2 males). ELSP’s assembled manchette microtubules (Supplemental Fig. S4, G-I; control spermatids, Supplemental Fig. S3) with NuMA labeling (Fig. 8L; Supplemental Fig. S4). Interestingly, rabbit anti-TACC3 did not detect manchette microtubules (Supplemental Fig. S4I) unlike a mouse monoclonal TACC3 antibody (Supplemental Fig. S3J-L). Microtubules were contiguous in the fused spermatid: Sertoli dyad, mostly within the cortex (Supplemental Fig. S4C and E, insets). However, Sertoli cell apical microtubules strongly labeled with aurora A kinase antibody, with microtubules interacting with round and elongating spermatids (Fig. 8M; Supplemental Fig. S4B). Aurora A kinase antibody was found on some ELSP centrosomes, select microtubules, sperm axonemes, and weakly labeling manchette microtubules (Fig. 8M, inset; Supplemental Fig. S4B). NuMA also detected in ELSP centrosomes, sperm axonemes, and labeled intact RS and SC nuclear proteins (Fig. 8J; Supplemental Fig. S4K and N). The molecular motor Eg5 was detected in disassembling manchette microtubules (Supplemental Fig. S4E and O) and RS acrosomal caps while KIFC1 bound ELSP centrosomal and apical heads (Supplemental Fig. S4C and F).

A dodeca-nuclear spermatid with incorporated SC enters spermiation having extensive ELSP cytoplasmic polarization (Fig. 8N-Q; Supplemental Fig. S5; 3/35; 9%; 3 males). Microtubules with multiple cytoplasmic γ-tubulin foci assembled from the apical SC, with single microtubules interacting with one ELSP nucleus and more extensive MT with multiple γ-tubulin foci around an immature ELSP (Fig. 8N, P, Q). However, γ-tubulin was not in spermatid implantation fossa (Fig. 8N, Q; compare Supple Fig. S5D). Curiously, multinuclear ELSP lacking SC association did not show extensive microtubule assembly or γ-tubulin (Supplemental Fig. S5F and H). TACC3 was absent from ELSP, despite TACC3 foci in SC and other spermatocytes (Supplemental Fig. S5D, F, and H, insets). Collectively, post-meiosis polyploid spermatids apparently continue cytodifferentiation like control cells i.e., sperm axoneme formation, acrosomal formation, centrosomal nuclear binding and positioning, nuclear cytoplasmic polarization, Sertoli cells interaction, and nuclear shaping by transient assembly of manchette microtubules. Late stage multinuclear ELSP might enter early spermiation in association with Sertoli cells, although this is highly speculative without proper functional assays. We also cannot address if multinuclear ELSP are developmentally competent without performing proper fertilization tests.

## Discussion

This study provides evidence of polynuclear spermatocytes and spermatids as a common feature of the normal testicular cell populations with possible developmental potential like normal control spermatocytes. Polynuclear spermatocytes and spermatids are distinct from multinuclear giant cells that are abnormal in phenotype, suffering a variety of nuclear and cellular defects with limited developmental potential that typically die. We speculate that polynuclear primary spermatocytes arise after mitotic spermatogonia errors, either by failing mitotic cytokinesis or when spermatogonia undergo cell-to-cell fusion while organized in long chains interconnected by intracytoplasmic bridges. Polynuclear secondary spermatocytes and spermatids also arise by failure of control spermatocytes to complete first or second meiotic cytokinesis. Polynuclear primary spermatocytes derived from spermatogonia errors appear to progress through critical pre-prophase-I stages and enter first meiosis to assemble functional multiple bipolar spindles of distinct phenotypes. Lacking the spindle pole protein KIFC1, meiotic polynuclear primary spermatocytes do not cluster their poles despite amplified centrosomes. We hypothesize that polynuclear primary spermatocytes progress through meiotic development without completing either first or second meiotic cytokinesis, resulting in single polynuclear spermatids post-meiosis. Remarkably, multinuclear spermatids are identified in many hallmarks of post-meiotic cytodifferentiation, forming elongated spermatids in association with Sertoli cells that may progress into early spermiation. While a retrospective analysis does not rule out other potential pathways for generating polynuclear spermatocytes or spermatids, we recognize that these cells are not unique in the transgenic GFP-CETN2-expressing mice, as multinuclear spermatocytes and spermatids are readily identifiable in male testicular samples from non-transgenic CB6F1 and C57BL mice.

Polynuclear primary spermatocytes are hypothesized to arise from mitotic spermatogonia proliferation errors, namely failed cytokinesis, or by cell fusion of spermatogonia connected by ICBs in a syncytial chain (Fig. 2)^4^. Additionally, polynuclear secondary spermatocytes or spermatids arise when single nuclear primary spermatocytes fail either first or second cytokinesis. We advance these hypotheses on polynuclear spermatocyte formation based on cellular diameters, nuclear counts and chromatin architecture after DNA dye staining, as well as centriole numbers in relation to known duplication stages^19^. Our methods avoid using thin histological testicular sections through the testis that have limited, difficult immunostaining issues or harsh chemical treatments that are incompatible with preserving delicate cytoskeletal architecture. Nevertheless, we investigate only that fraction of material release from fixed seminiferous tubules that adhere to our polylysine-coated coverslips. Furthermore, fixation chemicals and mounting coverslips onto slides can introduce errors on preserving accurate cell diameters or cellular architecture. It is possible that other pathways generate polyploid spermatocytes or post-meiosis spermatids. We have investigated short, ‘post-quashed’ partially depleted immunostained seminiferous tubules whole mounted for comparison to our isolated single cells to bolster our confidence in our findings reported here. Still, our interpretations are derived from ‘snapshots’ from limited sample sizes. A tractable dynamic in vitro model to follow the generation and fate of living polynuclear spermatocytes will be fascinating and awaits future development.

Regardless of polynuclear primary spermatocyte derivation, these unique cells appear to accomplish pre-prophase-I substage events, like homologous chromosome pairing with presume DNA crossover activity and first centriole duplication at leptonema, like reported in control spermatocytes (Fig. 2)^19^. Surprisingly, polynuclear primary spermatocytes assemble multiple spindles at metaphase-I with unique phenotypes depending on their supposed spermatogonia derivation pathway (Fig. 2). Why these unique spindle phenotypes arise differently in cytokinesis failures versus spermatogonia cell fusions is not fully clear. But combining two or more perhaps unrelated cell cytoplasm into a single spermatocyte seems to support independent bipolar spindle assembly in large di-nuclear, tri-nuclear, or greater polyploid spermatocytes (Fig. 1). This is further supported by observations that both small di-nuclear primary spermatocytes believed derived by mitotic cytokinesis error and normal spermatocytes that produce di-nuclear secondary spermatocytes after failing first meiotic cytokinesis both form multipolar spindles with more central aligning chromosomes (Figs. 2 and 5).

Polynuclear primary spermatocytes with amplified centrioles do not robustly assemble clustered bipolar spindles as observed in cancer cells (Fig. 3 and Suppl Fig. S1)^51^. Amplified centrosome clustering in cancer cells is complex involving interplay among microtubule motor proteins (dynein, KIFC1 [kinesin-14), nonclaret disjunctional [ncd] kinesin, Eg5 [kinesin-11]), and spindle associated proteins (NuMA, TACC3, cKAP5chTOG), among others^52^. Recently, spindle TACC3 was shown upregulated in certain cancers with poor clinical outcomes and its interaction with KIFC1 (HSET) vital to bipolar spindle assembly, as disruption of their interaction produced multipolar spindles and mitotic cell death^24,52,53^. TACC3 is present in spermatocytes meiotic spindle poles where, with the frequent binding partner cKAP5chTOG, helps spindle stabilization^19,20^. KIFC1 was reported in mouse spermatocyte meiotic spindles, with specifics inhibitors compromising spindle integrity, altering centrosome numbers and inducing aberrant chromosomal segregation^42^. Here, KIFC1 was not detected in polynuclear meiotic spindles, unlike in mitotic MCF7 cancer cells (Fig. 3) and post-meiotic spermatids (Fig. 8). Furthermore, a single cell RNA seq database of gravity sedimented adult mouse testicular cells showed robust distribution and density of TACC3 but not KIFC1 mRNA (Fig. 3, panel IV)^47^. Eg5 (kinesin-5) and NuMA, other critical spindle pole focusing proteins, are present in polynuclear meiotic spindles as in control spermatocytes (Fig. 3, panel III)^44,45^. Thus, KIFC1 absence in meiotic spindles of polynuclear spermatocytes may explain the lack of spindle pole clustering despite enhanced centrosome amplification. Neither Eg5 nor NuMA appear capable of compensating for KIFC1 deficiency. Interestingly, meiotic polynuclear spermatocytes appear to remain viable with multiple spindles or spindle poles without activating apoptosis pathways as found in some cancer cells^51^. Indeed, these findings are consistent with limited studies on meiotic spindle assembly checkpoint (SAC) in male mice that show more accurate chromosome segregation and lower SAC fidelity than in female oocytes^54^. How meiotic polyploid spermatocytes avoid or alter known meiotic checkpoint mechanisms is currently not understood.

Meiotic polynuclear primary spermatocytes assemble multiple bipolar spindles that can progress asynchronously, impacting early midbody assembly but not chromosome segregation (Fig. 4). We found that both small and large primary polyploid spermatocytes fail first cytokinesis despite multiple assembled midbodies/ICBs (Figs. 6 and 7). Additionally, control single nucleus primary spermatocytes occasionally fail first cleavage, forming bi-nuclear interkinesis spermatocytes or spermatids post-meiosis with a single midbody/ICB without cleavage (Fig. 5). Taken together, polynuclear spermatocytes arise from a deficiency in the cleavage furrow assembly machinery^3^, though the exact mechanism remains unresolved. In control single nucleus spermatocytes, the meiosis specific non-muscle myosin IIB isoform is essential for meiotic cytokinesis, although elegant genetic studies on the depletion of MYH10 gene in mouse spermatogenesis caused abnormal nuclear segregation phenotypes and cell cytodifferentiation arrest not readily apparent in our polyploid spermatocytes^55^. We did not investigate if meiotic polyploid spermatocytes midbodies convert to stable ICB’s that are critical to maintain germ cell synchronization during development^3^. It will be fascinating to explore modified sphingolipids for cleavage initiation and stabilization of the ICB^10,56,57^ or Testis Expressed protein 14 (Tex14) and the centrosomal protein CEP55, critical components in blocking abscission in meiotic diploid spermatocytes^3,58^, in polyploid meiotic spermatocytes.

Polynuclear primary spermatocytes enter interkinesis and appear to initiate second centriole duplication (Figs. 5 and 6)^19,59^. At metaphase-II, polynuclear secondary spermatocytes assembled multiple spindles with phenotypes mirroring those observed in first meiosis (Figs. 2 and 5, and 6). Polynuclear secondary spermatocytes also fail second cytokinesis after completing successful chromosome segregation (Figs. 5 and 6, and 7). Importantly, we confirmed assembled metaphase-II spindles had correct GFP-CETN2 expressing centriole numbers at their poles, consistent with normal meiotic progression as opposed to an aberrant spindle assembly event (Figs. 5 and 6).

Post-meiosis polyploid spermatids were identified with sperm axonemal formation, sperm centrosomal nuclear association, and Golgi/acrosome assembly (Fig. 8). Sperm axoneme assembly is a complex multi-step event that begins early in mouse spermiogenesis steps 2–3 at the distal centriole^49^. We identified early sperm axoneme assembly by differential interference contrast (DIC; Fig. 8B, arrowhead) and lengthening axonemes with docked centrosomes to nuclear surfaces at step 4–7, a critical event for axonemal generation (Fig. 8, F; Supplemental Fig. S2)^60^. Axonemal development in polynuclear spermatids continued through early suspected spermiation (Fig. 8, N-Q; Supplemental Fig. S5), although specific hallmark events in axonemal construction await further study. Transient microtubule labeling at early acrosomal caps was observed in polynuclear spermatids (Fig. 8a-E), like control round spermatids (Supplemental Fig. S3, A-C)^48^. TACC3, but not cKAP5chTOG^61–63^, labeled likely Golgi (Fig. 8, G-I; Supplemental Fig. S2). We speculate that acrosomal cap microtubules, KIFC1 (Supplemental Fig. S4, F) and TACC3 might be involved with Golgi proacrosomal vesicles translocation to nascent nuclear acrosomal vesicles^64^.

Manchette microtubules, first detected at step 8 in mice, are crucial for nuclear condensation and elongation during mouse spermatid development^49,65^. Manchette microtubules assemble in polynuclear ELSP (Supplemental Fig. S4, G-I), like control spermatids (Supplemental Fig. S3). Interestingly, NuMA microtubule binding protein^64,66^ assembled on polyploid spermatid manchette microtubules towards the sperm axoneme/basal body region, with weaker NuMA possibly at the perinuclear ring (Fig. 8, L), perhaps participating in manchette microtubule assembly, dynamics and/or nuclear head shaping. Intranuclear NuMA was detected in round spermatid and Sertoli cell nuclei (Supplemental Fig. S4, K and N). Step 13–14 polynuclear spermatids appear to disassemble Manchette microtubules prior to sperm midpiece assembly, like observations in step 13–14 control spermatids^49^, with NuMA restricted to basal centrioles and the progressing sperm axoneme (Supplemental Fig. S4, K and N).

Eg5 (KIF11), a plus-end directed bipolar spindle microtubule motor protein required for proper spindle assembly, stabilization, and microtubule flux is phosphorylated by multiple Src kinases in mitotic somatic cells^67^. Eg5 (KIF11) has been reported in mouse mitotic spermatogonia and meiotic spermatocytes but not spermatids or Sertoli cells^68^. Here, Eg5 staining localized in disassembling Manchette microtubules, restricted to the sperm basal region by steps 13–16 (Supplemental Fig. S4 E, L, and O). Aurora A kinase, which regulates many M-phase proteins including NuMA^66,69^ was found in a subset of manchette microtubules, especially along the basal sperm head region, and in elongating spermatid centrosomes and axonemes (Fig. 8, M; inset, single sperm head; Supplemental Fig. S4, B). Sertoli cell Aurora A-labeled microtubules interact with aberrant round spermatid nuclei, perhaps assisting in their elimination (Supplemental Fig S4, B).

Spermiation beginning at step 16 (Stage VII) in mice is the complex multistep process involving the “spermiation machinery” for completing elongated spermatid remodeling, epithelium positioning and disengagement into the lumen^4^. Multinuclear spermatids with contiguous integrated Sertoli cells in a common cytoplasm were observed in suspected early spermiation stages (Fig. 8, O; Supplemental Fig. S5, C-D). This configuration showed polarized mature ELSP opposite the Sertoli cell and apical Sertoli cell assembled microtubules with multiple γ-tubulin foci interacting with spermatids (Fig. 8, P, arrow). Sertoli cell microtubules appeared significantly concentrated around immature spermatids (Fig. 8, Q). Late spermatid maturation includes shedding residual bodies, the extra cytoplasm from spermatids which contains many sperm proteins constituents including γ-tubulin^21,70^. The lack of sperm γ-tubulin at the implantation fossa suggests shed residual bodies (compare, Fig. 8, N and Q with Supplemental Fig. S C-D), but requires additional confirmation. Interestingly, late-stage polynuclear spermatids without integrated Sertoli cells had poor epithelial microtubule organization with scant sperm or cytoplasmic γ-tubulin, despite showing ELSP polarization and advanced sperm axonemal development (Supplemental Fig. S5, E-H). TACC3 was not found in late-stage spermatids despite labeling in adjacent spermatogenic cells (Supplemental Fig. S5, inset). Thus, multinuclear spermatids probably enter the final maturation stages under the direction of Sertoli cells, consistent with preparation for disengagement into the lumen. However, this still needs to be confirmed and will be a fascinating area for future investigation.

In summary, evidence presented here shows the natural occurrence of polynuclear spermatocytes and spermatids among the tightly organized seminiferous tubule population of germ cells and somatic testicular cells in mice. Polynuclear primary spermatocytes probably arise from errors in proliferating spermatogonia and secondary spermatocytes from meiotic cytokinesis failures. Polynuclear spermatocytes appear viable and distinct from multinuclear giant cells commonly seen in testis cells after mouse genetic manipulations or chemical/physical insults. Like normal control spermatocytes, polynuclear spermatocytes can be identified at key first and second meiotic stages, despite assembly of multiple meiotic spindles with amplified centrosomes that lack KIFC1 microtubule motor clustering protein, a condition that equates with cell death machinery activation in many cell types. Post-meiosis, polynuclear spermatids are identified along key cytodifferentiation steps and appear to enter spermiation. This study provides an important identification of a subset of derived testicular cells that can provide important clues regarding generation of testicular sperm production and fertility. It may also provide interesting clues to understanding the molecular underpinnings of spermatocyte cell cycle control for designing novel targets for male contraception.

## Methods

### Mouse husbandry, handling, and institutional oversight

All animal procedures were approved by the Institutional Animal Care and Use (IACUCs) Committees at the University of Pittsburgh and Magee-Womens Research Institute (protocol #22010530) in compliance with the National Institute of Health’s Office of Laboratory Animal Welfare *Guide for the Care and Use of Laboratory Animals* and the ARRIVE guidelines. CB6-Tg (CAG-EGFP/CETN2)3-4Jgg/J mice (Stock number: 00823445) were obtained from the Jackson Laboratory (Bar Harbor, ME) as juveniles, bred, and analyzed as described previously^20^. All mice were housed in an Association for Assessment and Accreditation of Laboratory Animal Care (AAALAC)-accredited mouse facility and tissues collected after humanely euthanizing mice by carbon dioxide (CO_2_) asphyxiation (55–60 cubic feet per hour flow rate; 2–4 min) followed by cervical dislocation using approved methods by the American Veterinary Medical Association (AVMA) and our University-approved IACUC protocols as described previously^20^. Tissues were harvested within 5 min post-euthanasia and fixed within ~ 1.5 h post-harvest. Mice ages ranged from 6 to 18 months and Weighed between 32 and 36 g.

### GFP expression determination by PCR

Genomic DNA was isolated for GFP detection in GFP CETN2-expressing mice, using tail tip tissues (< 5 mm) and PCR with MyTaq Extract-PCR Kit (Bioline, Taunton, MA) as previously described^25^.

### Spermatogenic cell isolation from fixed seminiferous tubules

Seminiferous tubules (ST) from GFP CETN2-expressing male testes were collected after euthanasia as previously described^20^. Briefly, excised testis was placed in Minimum Essential Medium α (MEM α) containing 10% KnockOut™ Serum Replacement (KSR) and 1000U/ml and 1000 µg/ml Penicillin-Streptomycin (P/S) (components from Thermo Fisher, Waltham, MA) for mechanical removal of the tunica albuginea and fat pad Material. After a brief incubation in 3 ml of 1 mg/ml collagenase IV in αMEM (no KSR or P/S), seminiferous tubules were Manually cut into ~ 20-mm-long pieces with sterile scissors, washed twice in αMEM/10%KSR/1%P/S by gravity sedimentation, and then fixed in 0.5% paraformaldehyde (pFA) in αMEM (no KSR or P/S) for 30 min at 34 °C. After fixation, fixed tubules were then Transferred to a 2-well chamber slide (Nunc; Thermo Fisher). Using a vacuum plunger (Pen-Vac; Ted Pella, Redding, CA) to hold an 18-mm plastic coverslip (Rinzl; Electron Microscopy Sciences, Hatfield, PA), pressure was applied to squash the fixed ST and release fixed cells as described^22^. Released cells and small ST pieces were harvested from the wells using a p1000 pipet with tip cut to prevent damage, and cells/tissue were placed in the center of a 22-mm^2^ poly-lysine-coated slide (2 mg/ml; Sigma-Aldrich) that had been rinsed with distilled water and 0.25% Triton X-100 in PBS (PBS-TX). After 5 min, cells were permeabilized in PBS + 2% (PBS-TX) for 30 min before further processing for immunocytochemistry staining as described below.

*Spermatogenic cell isolation from seminiferous tubules using enzymatic methods* was performed as previously described^20^. Briefly, ST were Manually cut into ~ 20-mm-long pieces and incubated in 5 ml of 0.05% trypsin:0.53 mM EDTA (Thermo Fisher) for 20 min with rocking agitation in a 37 °C/5% CO_2_ incubator as previously noted^20^. The trypsin: EDTA solution was neutralized with an equal volume of αMEM/1% KSR/1% P/S and the cell suspension passed through a 100-micron cell strainer (Fisher Scientific, Pittsburgh, PA) into a 50-ml Falcon tube, 10mL of αMEM/1%KSR/1%P/S was added, the cell suspension centrifuged at 125 x g for 3 min at room temperature to pellet testicular cells. After a brief wash in 1mL αMEM (no KSR or P/S), the cell suspension was centrifuged again at 125 x g for 3 min at room temperature. The supernatant was gently removed and pelleted cells plunged into 100% cold MeOH for 5 min at −20 °C. Fixed cell suspension were centrifuged twice at 125 x g for 3 min at room temperature before resuspending the final cell pellet is in 1.5-2mL of PBS + 0.25% Triton, attaching fixed cells to polylysine-coated coverslips and processing for immunostaining as presented below.

### Primary mouse embryonic fibroblasts and MCF 7 human breast cancer cell line culture, lentiviral transduction with CETN2-GFP, fixation and immunostaining

Commercially available primary mouse embryonic fibroblasts isolated from CF-1 mice (Millipore; catalog # PMEF-CF) and human breast cancer cell line, MCF7, (ATCC, Manassas, VA; catalog # HTB-22) were thawed and propagated using protocols according to the manufacturer and previously described^28^.

For transduction with lentiviral CETN2-GFP (a generous gift from Dr. Jeffrey Salisbury; Mayo Clinic, Rochester, MN), viral particles were produced using the ViraPower Lentiviral Packaging Mix (Thermo Fisher) according to the manufacturer’s recommendations and previously described^20^. Briefly, CF-1 primary mouse embryonic fibroblast cell lines were transduced after plating cells into a T-25 cell culture-treated flask at 37 °C overnight with viral particles and 6 mg/mL of polybrene (Millipore). After 24 h., transduced cells were washed once with PBS, and transduction medium was replaced with Feeder Cell Complete cell culture medium. Confirmation of GFP-CENT2-expressing centrioles was confirmed in cells seeded overnight on sterile 22-mm^2^ coverslips and fixed in 1% paraformaldehyde in DMEM without protein for 40 min at 37 °C. Immunostaining was performed as described below.

### Immunocytochemistry

Coverslips containing fixed spermatogenic cells, short ST pieces or tissue culture cells were first permeabilized in 2% PBS-TX for 30 min at room temperature before blocking in BlockAid (Thermo Fisher) for 40 min at room temperature as described previously^20^. Details on primary antibody utilization and characteristics, including Research Resource Identifiers (RRID; https://scicrunch.org/resources/about/guidelines), are presented in Supplemental Fig S1. As previously described^20^, all primary antibodies were diluted to final concentrations in sterile PBS and applied overnight at 4 °C. Primary antibodies were detected with appropriate fluorescently tagged secondary antibodies (ThermoFisher) at room temperature for 2 h. in the dark after PBS rinses. DNA (final concentration of 10 µg/ml, each) was labeled with a combined DNA stain of Hoechst 33,342 and DAPI from a 1 mg/ml stock solution for 10 min at room temperature before mounting in Vectashield antifade mounting medium (ThermoFisher) and sealing with nail varnish.

### Single-cell transcriptomic analysis

Mouse single-cell RNA-seq datasets were downloaded from the Mendeley Data (data.mendeley.com, DOI: 10.17632/kxd5f8vpt4.1) as queryable and annotated files stored in Loupe Cell Browser files (.cloupe) (10X Genomics, Loupe Browser, v8.0.0)^47^. Mouse unselected three replicates of steady-state spermatogenic cells, 4,651 cells), mouse sorted (Aggregated Ad Spg- ID4-EGFP bright/dim/CD9bright, 6,945 cells), and mouse StaPut spermatocytes (4,233 cells) were analyzed for KIFC1 and TACC3 expression. Single-cell data were presented in t-Distributed Stochastic Neighbor Embedding (t-SNE) plots with annotations assigned by the authors of the study^43^. The expression levels were presented in log2-transformed unique molecular identifier (UMI) count overlayed on t-SNE plot and violin plot clustered for the selected cell types.

### Equipment, Imaging, analysis, and settings

Imaging fixed slides was accomplished as described by Simerly et al.^20^. Briefly, images were collected with a Nikon A1 four-laser line confocal microscope equipped with Elements A1 Plus compact GUI acquisition software (version 4.20; Nikon USA) at 1024 × 1024 size at ¼ frame per second, using a pinhole size of 79.2 mm and a z-depth of 0.25 mm through the entire specimen, with a differential interference contrast (DIC) Plan Fluor x100 (1.4 NA) objective. We collected 5 × 12-bit depth images (nd2 files), using the same laser photomultiplier tube settings for each channel across specimens (5% laser power, except UV, for DNA imaging, at 10.24%) to facilitate fluorescent intensity ratios, surface intensity plots, areas, or volume measurements. Image analysis was performed on binarized images, using the threshold tool and region-of-interest statistical menu in the Elements software, and downloaded to Microsoft Excel for statistical analysis. For image panel presentation, generated confocal nd2 files were first denoised in the A1 software before performing a subtracted background image, collected from outside of the specimen field. All channels were then subjected to the deconvolution software module in Elements (Landweber algorithm) using the point-scan confocal command and same filter (noisy) at twenty iterations for all images. Final panels from deconvolved images were prepared in Photoshop (Adobe Systems, San Jose, CA). All images panels reflect typical observations for each stage of polynuclear spermatocytes or spermatids collected and analyzed by confocal microscopy. Rare observations are noted in the text.

*Statistics* were performed as described previously^20^. Means ± standard deviations were determined using calculator.net (Maple Tech International, LLC; The Woodlands, TX). We used Excel (Microsoft; Redmond, WA) to prepare graphs and box plots, which show median (horizontal lines), 25th and 75th percentiles (small boxes), and min and max (whiskers). Statistical significance was determined by Student’s T-test (GIGA calculator; Web Focus, LLC; Sofia, Bulgaria). Significance was determined at *p* < 0.05. Graphical analyses shown are indicative of average values ± standard deviation. Data are representative of all trials.

## Supplementary Information

Below is the link to the electronic supplementary material.

Supplementary Material 1

## Data Availability

Datasets generated and/or analyzed during this study are available from the corresponding author on reasonable request as set forth in the guidelines of this journal.
