# Supplementary material for: Meiotic progression in multinuclear mouse spermatocytes without the spindle pole clustering motor protein KIFC1 or cytokinesis forms single-cell late-stage spermatids
Source: Sci Rep. 2025 Oct 17;15:36399. doi: 10.1038/s41598-025-20463-2 (PMC12534425; doi:10.1038/s41598-025-20463-2)
Supplement: Supplementary file 1 — Supplementary Material 1 [file 41598_2025_20463_MOESM1_ESM.pdf]

**SUPPLEMENTAL MATERIAL**.

# Meiotic Progression in Multinuclear Mouse Spermatocytes without the Spindle Pole Clustering Motor Protein KIFC1 or Cytokinesis Forms Single-Cell Late-Stage Spermatids.

by

Calvin Simerly[1], Carrie Hartnett[1], Ashley Zyhowski[1] Emily Robertson, Caleb Harrison, In Ki Cho[2], Charles Easley IV[2], and Gerald Schatten[1,3]

[1]Pittsburgh Development Center of Magee-Womens Research Institute, Departments of Cell Biology, Ob-Gyn-Repro Sci, and Bioengineering, University of Pittsburgh Medical Center, 204 Craft Avenue, Pittsburgh, PA 15213; [2]Environmental Health Science and Regenerative Bioscience Center, College of Public Health, Edgar L. Rhodes Center for Animal and Dairy Science, 425 River Rd, Athens, GA 30602.

[3]corresponding author; gschatten@pdc.magee.edu

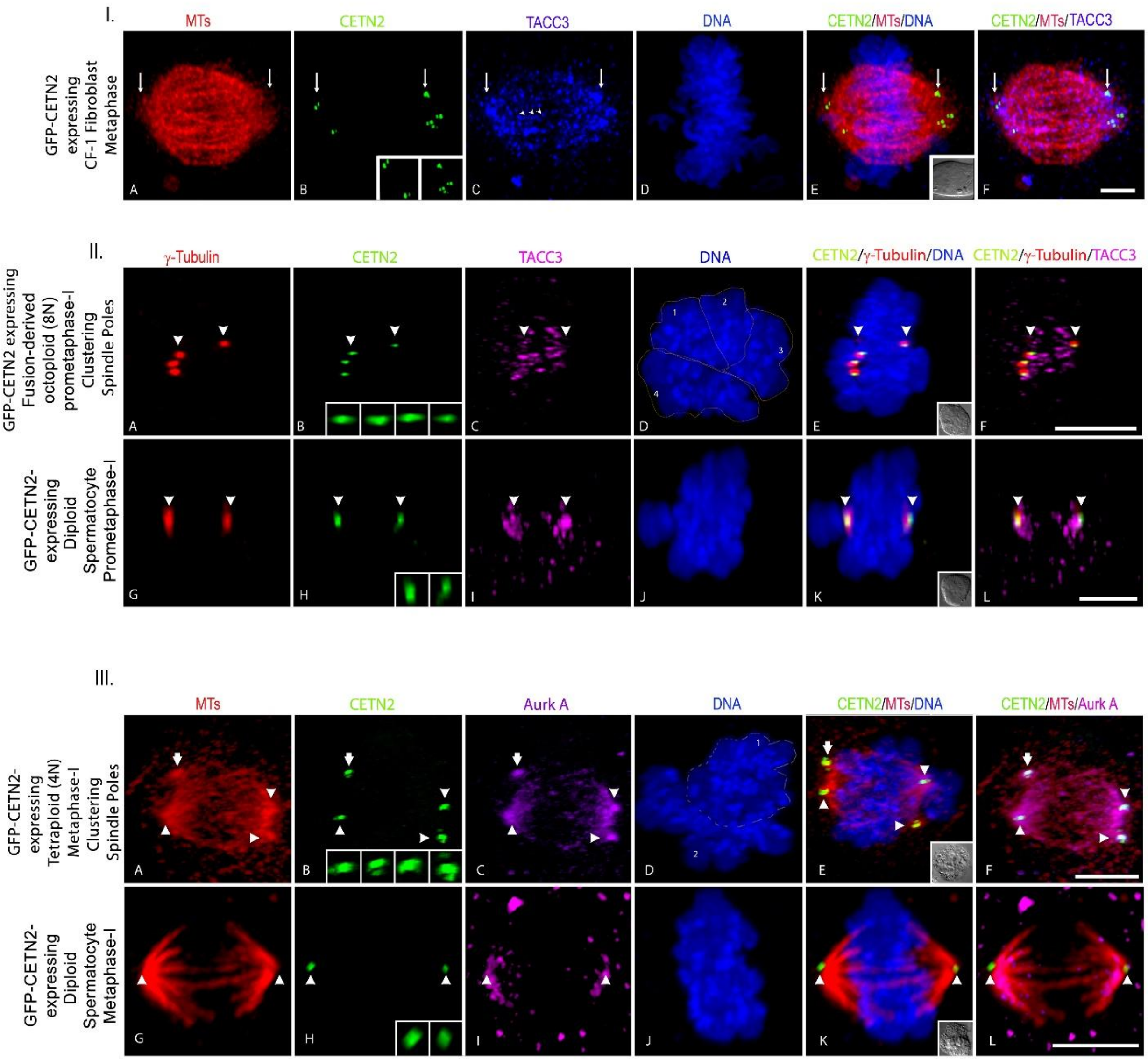


**Supplemental Figure S1**. **Meiotic-I polynuclear primary spermatocytes with clustered bipolar spindles.** *Panel 1*.**A-F:** CF1 fibroblast cell with amplified GFP-CETN2 expressing centrioles (B: green, arrows; insets, details) clustered on a bipolar metaphase spindle (A: red, microtubules) with slightly misaligned chromosomes (D: DNA, blue; E, F: overlays). E: inset, DIC. Scale bar: 5 µm.

*Panel II*. **A-F***:* Large quad-nuclear GFP-CETN2-expressing primary spermatocyte at prometaphase-I showing 4- γ-tubulin foci (A: red) surrounding 4 centriole doublets (B: green; inset, details) split unequally between two spindle poles (A-C: arrowheads; E:

overlay; inset, DIC). TACC3 (magenta) is dispersed onto short spindle pole microtubules and centrosomes (F: overly, arrowheads). D: blue, DNA; chromosome sets 1→4. Estimated cell diameter=23 μm.

**G-L**: Single nucleus GFP-CETN2-expressing prometaphase-I primary spermatocyte with two $\gamma$-tubulin foci (G: red; arrowheads) at opposite spindle poles with centriole pairs (H: green; arrowheads) and slightly unaligned chromosomes (J: blue, DNA; K: overlays; inset, DIC). TACC3 (I: magenta, arrowheads) concentrates at the spindle pole centrosomes (L: overlays). Estimated cell diameter: 14 μm. Scale bars= 5 μm.

*Panel III*. **A-F:** Large di-nuclear GFP-CETN2-expressing metaphase-I primary spermatocyte showing a broad anastral bipolar spindle (A: red, microtubules, arrowheads) with 4 distinct spindle pole centriole doublets (B: green, arrowheads; short arrow slightly separated centriole doublet) but unaligned chromosome sets (D: blue; E: overlay; inset, DIC). The TACC3 phosphorylating kinase Aurora A (Aurk A) is located on meiotic spindle poles, including the centrioles (C; magenta, arrowheads; short arrow: duplicated centriole doublets). Estimated cell diameter= 25μm. **G-L**: GFP-CETN2-expressing control metaphase-I spermatocyte with a bipolar spindle (G: red; microtubules; arrowheads label spindle poles), spindle pole centriole doublets (H: green, arrowheads; insets; details), and DNA aligned at the spindle equator (J: DNA, blue; K: overlay; inset, DIC). Aurora A kinase detection is tightly localized to the centrosomes and spindle pole microtubules (I: magenta; arrowheads mark centrioles; L: overlay). Cell diameter: 14 μm. Scale bars= 5 μm.

| Sample | Total population n (%) | Derivation Stage | n observations (%) |
|---|---|---|---|
| Control Meiotic Spermatocytes | 33/933 (4) | Cytokinesis-I Failure; dual interkinesis nuclei; either 2 (pre-duplication) or 4 (post-duplication) centriole doublets | 19/33 (58) |
| Early Spermatids | | Cytokinesis-I and II Failures; 4- Round Spermatid Nuclei; 4-centriole doublets | 8/33 (24) |
| Early Spermatids | | Cytokinesis-II failure; dual Round Spermatid Nuclei; 2- centriole doublets | 6/33 (18) |
| **Primary Polynuclear Spermatocyte type** | **Total population n (%)** | **Spindle Phenotype** | **n observations (%)** |
| Small Di-Nuclear | 29/183 (16) | multipolar; central chromosome alignment | 23/23 (100) |
| Large Di-Nuclear | 111/183 (61) | independent dual-spindle assembly each with chromosome alignment | 49/82 (60) |
| | | tri-polar spindles each with chromosomal alignment | 30/82 (37) |
| | | back-to-back dual spindles, each with chromosomal alignment | 3/82 (3) |
| Large Tri-Nuclear | 36/183 (20) | Independent Tri-spindles, each with chromosomal alignment | 9/20 (45) |
| | | Single or dual adjacent spindle poles, independent chromosomal alignment | 6/20 (30) |
| | | Polyhedron spindle (1 pole for 3- spindles), independent chromosomal alignment | 5/20 (25) |
| Large, ≥ 4 Nuclei | 4/183 (2) | Multiple Independent Spindles with aligned chromosomes | 2/4 (50) |
| | | Multiple spindles, shared poles; each with aligned chromosomes | 2/4 (50) |
| Di-Nuclear Primary Spermatocytes with clustered Metaphase-I spindle poles | 3/183 (<2) | bipolar; mis-aligned central chromosomes | 3/3 (100) |

**Supplemental Table 1. Summary of Polynuclear Spermatocytes Derivation in Control Spermatocytes and Metaphase-I Spindle Phenotypes in Polynuclear Primary Spermatocytes.** Control spermatocytes fail first and /or second meiotic cytokinesis to generate polynuclear spermatocytes or early spermatids with varying nuclear and centriole numbers (rows 2-4). Polynuclear primary spermatocytes may form after defects in mitotic spermatgonia. Small di-nuclear primary spermatocytes derived after spermatogonia cytokinesis failure could produce multipolar spindles with more centrally aligned chromosomes (row 6). Conversely, spermatogonia in syncytial chains may undergo cell-to-cell fusion to produce large di-, tri- or greater nuclear polyploid primary spermatocytes. These polyploid primary spermatocytes appear to assemble varying first meiotic spindle phenotypes in a common cytoplasm (row 7-14). Regardless of how polynuclear primary spermatocytes form, clustering multiple spindle poles into a bipolar spindle apparatus is rarely observed (row 15).

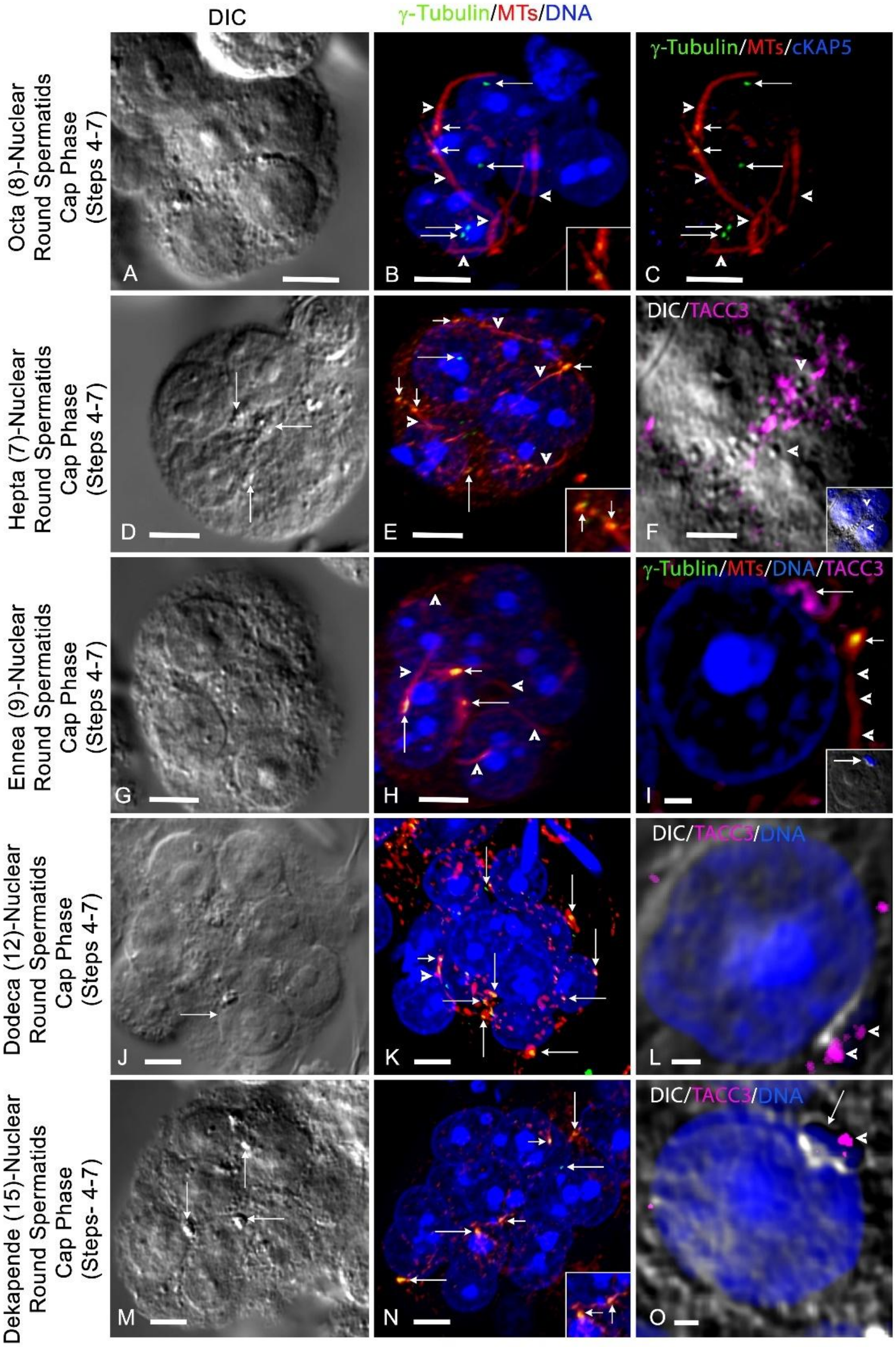


**Supplemental Figure S2. Post-meiosis polynuclear spermatids in early acrosomal cap stage (steps 4-7).** A-C: octoploid spermatocyte (A: DIC) with multiple sperm axonemes (B, C: red, microtubules, arrowheads) and γ-tubulin foci (green, arrows) on spermatid nuclear surfaces (B: blue, DNA) or sperm axonemal ends (B, C: short arrows; B, inset, details). Centrosomal

cKAP5chTOG is not apparent (C: blue). D-F: Heptaploid multinuclear spermatid with assembling acrosomal caps (D: DIC, arrows). Multiple cortical sperm axonemes (E: red, microtubules, arrowheads) are visible with $\gamma$-tubulin (E: green, short arrows; inset, details) or on nuclear surfaces (E: green, arrows). TACC3 (F: magenta) localizes to assembling acrosomal caps (F: DIC, arrowheads; inset, details; DIC and DNA, blue). G-I: Ennea (9)- nuclear stage spermatid (G: DIC). Cytoplasmic sperm axonemes (red, microtubules, arrowheads), some with $\gamma$-tubulin at the axonemal ends (H: green, short arrows) or on nuclei (H: green, arrows; DNA, blue). I: single nucleus (blue, DNA) with a sperm axoneme (I: red, microtubules, arrowheads), $\gamma$-tubulin (I: green, short arrow) and acrosomal cap TACC3 labeling [I: magenta, long arrow; inset, overlay of DIC and TACC3 (blue)]. J-L: dodecaploid polynuclear spermatid with assembling acrosomal caps (J: DIC, arrow). $\gamma$-Tubulin (K: green, arrows) is present on nuclear surfaces (DNA, blue) within small microtubule asters (K: red) and at least 1 sperm axoneme (K: green, short arrow; red, axoneme, arrowhead). L: single nucleus (L: DIC; DNA, blue) with acrosomal TACC3 (L: magenta; arrowheads). M-O: dekapende (15N) round spermatid with assembled Golgi (M: DIC, arrows). Short sperm axonemes are visible (N: red, microtubules; green, $\gamma$-tubulin, short arrows) with multiple nucleoli in formed nuclei (N: DNA, blue), indicating nascent spermatid status. $\gamma$-Tubulin is not present at the nuclear surface (N: green, long arrows). O: single nucleus (DIC; DNA, blue) with Golgi surface TACC3 (magenta, arrowhead) near the nuclear surface (O: long arrow). All images are from non-GFP-CETN2-expressing CB6F1 controls. B, C, E, H, I, K, N: $\gamma$-tubulin (green:), microtubules (red) and DNA (blue). C: cKAP5chTOG; F, I, L, O: TACC3 (magenta). DIC: differential interference contrast. All scale bars= 5µm except I, L, and O: 1-µm.

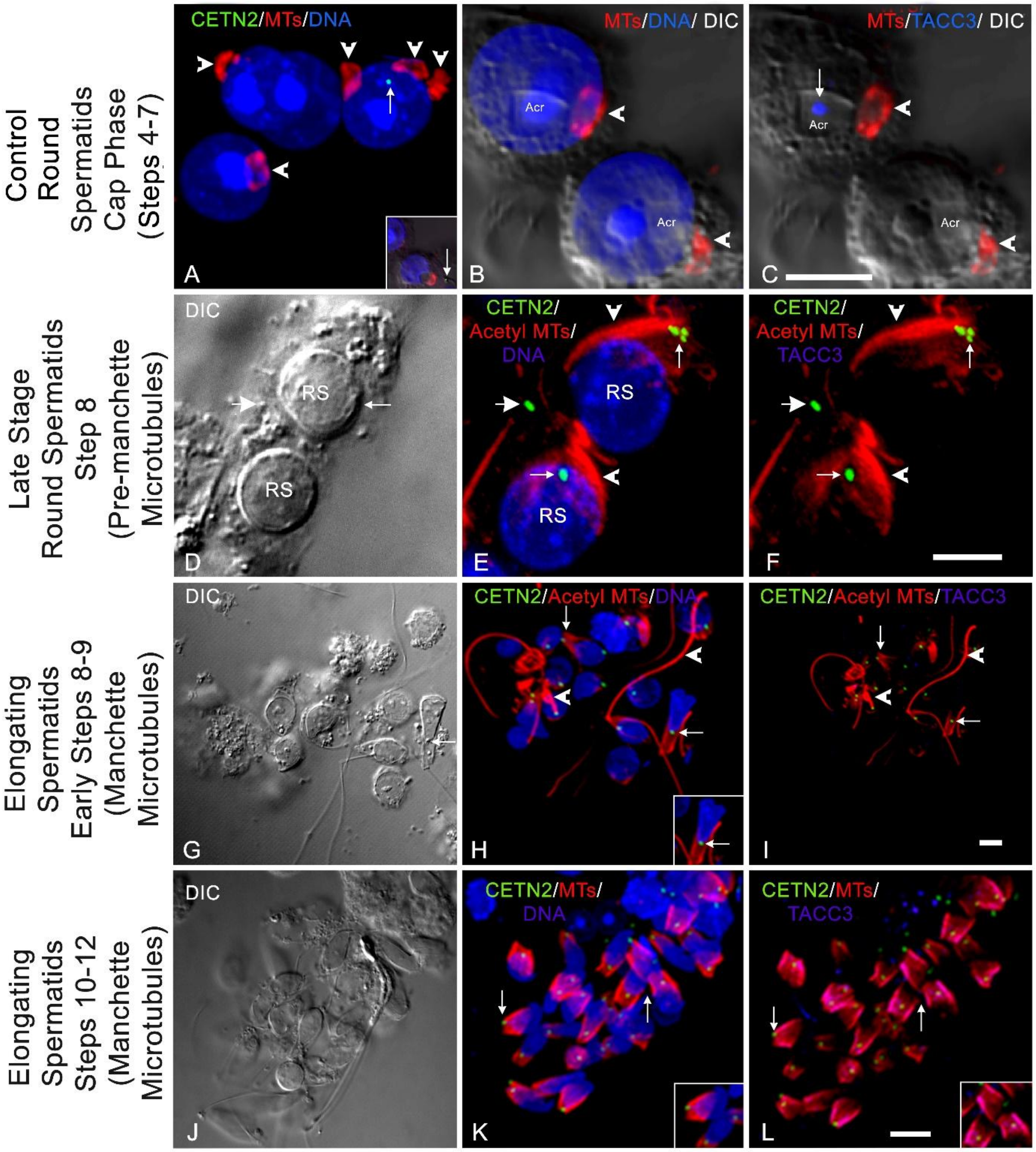


**Supplemental Figure S3. Post-meiotic diploid spermatocytes to early elongating spermatids stages.** A-C: Four early diploid round spermatids showing microtubule assembly at the developing acrosomal caps (red, microtubules, arrowheads). Round spermatids silence GFP-CETN2-expression early (A: green) with only a single GFP-CETN2 expressing centriole localized to the end of an axoneme in one spermatocyte [A: green, arrow; inset: overlay of DIC,

DNA (blue) and CETN2 (green showing centriole sperm axoneme localization (arrow)]. B: overlay of DIC, DNA (blue) and microtubules (red, arrowheads); Acr: acrosomal vesicle. C: overlay of DIC, microtubules (red, arrowheads) and TACC3 in an acrosomal vesicle (blue, TACC3, small arrow). D-E: late-stage diploid round spermatids showing cytoplasmic nuclear polarization (D: DIC, arrow), expression of GFP-CETN2 centrioles (E, F: green, arrows; DNA, blue) with strong acetylated tubulin detection of sperm axonemes (E, F: red, Acetyl Tub, arrowheads). TACC3 is not detected (E: blue) in late spermatids. D-E: small arrow, centriole at base of a sperm axoneme not associated with round spermatids. G-H: early diploid elongating spermatids (G: DIC; arrow; step 8-9) with GFP-CETN2-expressing centrioles (H, I: green, arrows; blue, DNA) and acetylated microtubules detected in manchette microtubules (H, I; red; inset, details) and some round spermatid axonemes (H, I: red, acetyl MTs, arrowheads). TACC3 detection (rabbit polyclonal antibody) was not observed in manchette microtubules (I: blue). J-L: later stage (step 10-12) elongating spermatids (J: DIC) showing assembled manchette microtubules (K, L: red, microtubules, arrows; K, inset: details) with robust GFP-CETN2-expressing centrioles (K, L: green; DNA, blue). L: TACC3 detection (mouse monoclonal antibody; blue, arrows) co-stains manchette microtubules (red; GFP CETN2 centrioles, green; inset, details). All images are GFP-CETN2-expressing spermatids co-stained with microtubules (A, B, C, K, L) or acetylated α-tubulin (E, F, H, I), DNA (A, B, E, H, K) and TACC3 (C, F, I, L). DIC: differential interference contrast. Scale bars= 5-μm.

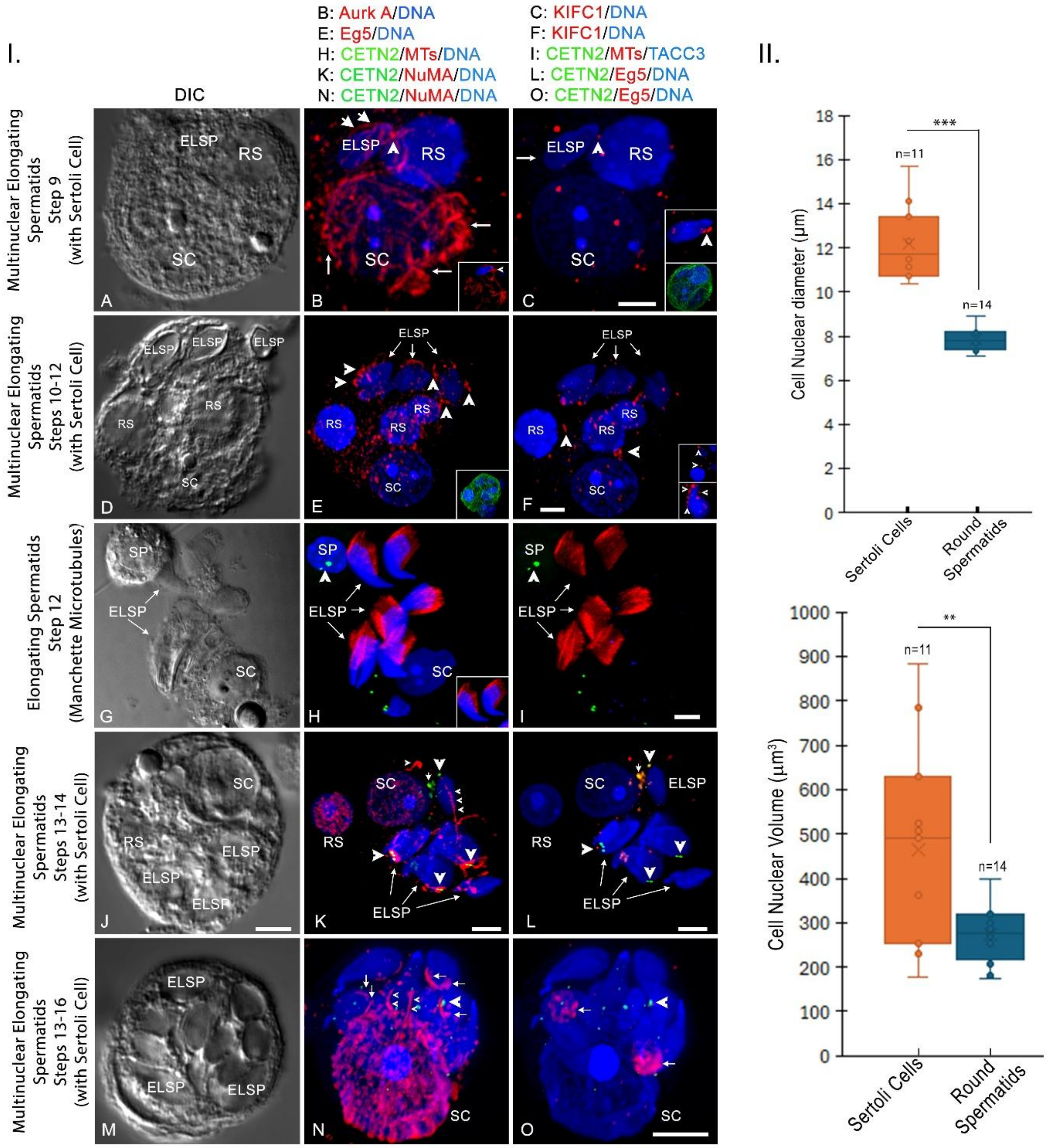


**Supplemental Figure S4. Multinuclear spermatids develop asynchronously with assembled manchette microtubules and Sertoli cell association.** *Panel 1.* A-C: a step 9 binuclear spermatid with a round spermatid (RS), elongating spermatid (ELSP), and a fused Sertoli cell (SC). A: DIC; B: anti-aurora A kinase microtubule detection in SC (B: red, arrows; blue, DNA) and ELSP centrosome/sperm axonemes (B: red, arrowhead and short arrows; inset, details). SC AurK A-labeled microtubules interact with the unhealthy RS (blue, DNA). C: KIFC1 labels the ELSP centrosome [red, arrowhead; upper inset, details; lower inset: cortical

microtubules (green) and DNA (blue)]. D-F: multinuclear step 10-12 spermatid with RS, ELSP and attached SC. ELSP are polarized, opposite from incorporating SC (D: DIC). Eg5 detects ELSP manchettes (E: red, microtubules, arrowheads) but not RS or SC. Inset: cortical microtubules (green) and DNA (blue). KIFC1 is concentrated at RS acrosomal caps (F: red, arrowheads; upper inset, details, arrowheads) and circumscribes ELSP nuclei (F: red, arrows; lower inset, arrowheads). G-I: Heptanuclear step 12 GFP-CETN2-expressing spermatid with ELSP and a SC (G: DIC). ELSP manchettes (H: red, microtubules, arrows; inset, details; blue, DNA) label but not centrioles (green). SP: a GFP-CETN2- expressing interkinesis spermatocyte (H: green, centrioles, arrowhead; DNA, blue). I: No rabbit anti-TACC3 (blue) is on ELSP manchettes (red, microtubules, arrows). J-O: two multinuclear step 13-16 spermatids with RS, ELSP and incorporate SC. J, M: DIC. Centrioles (K, N: green), NuMA (K, N: red, arrowheads) and Eg5 (L, O: red). NuMA transiently labels ELSP centrioles, disassembling manchettes (K, N: red, arrowheads), sperm axonemes (K: small arrowheads) and intranuclear NuMA in RS and SC nuclei (K, N: red; blue, DNA), but not SC centrioles (K: small arrow). Additionally, SC NuMA-labeled microtubules connect with ELSP (N: red, small arrowheads; DNA, blue). ELSP Eg5 is transient (O: red, arrows), though not at centrioles (L, O: green, arrowheads). Scale bars=5-µm. *Panel II.* Measured nuclear cell diameters (top graph) and volumes (lower graph) between Sertoli cells (orange bars) and round spermatids (blue bars). SC have greater nuclear diameters (****; $p < 0.0001$) and volumes (**; $p < 0.0071$) compared to diploid RS.

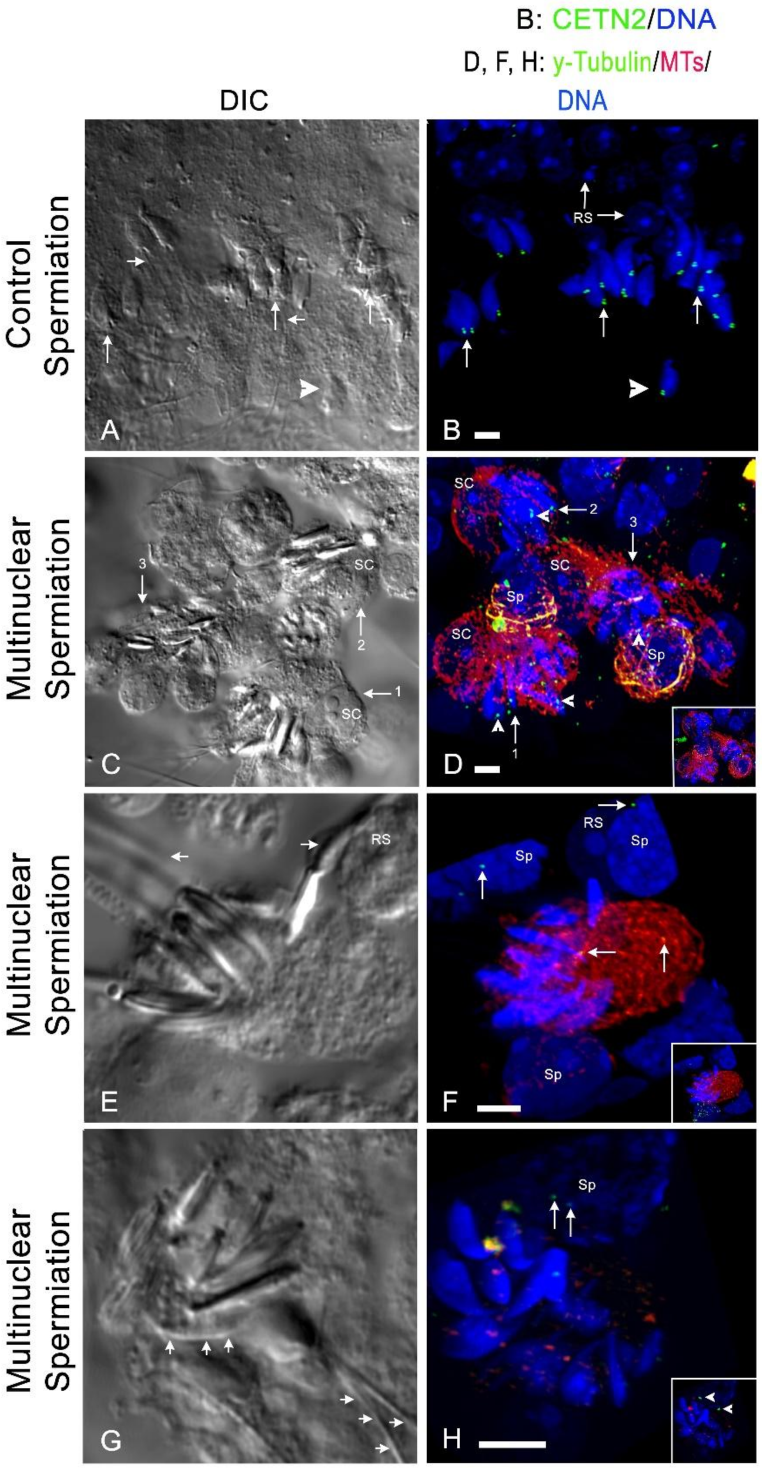


**Supplemental Figure S5. Multinuclear spermatids during spermiation.** A: GFP-CETN2-expressing late-stage diploid spermatids (A: DIC, arrows), one near the lumen (A: DIC, arrowhead), with attached sperm tails (A: DIC, short arrows). B: Image of GFP- CETN2 centriole doublets at the base of late spermatids (B: green, arrows; DNA, blue) in late spermatids nearing

spermiation. RS: round spermatids (arrows). C: DIC: area showing three late-stage multinuclear spermatid clusters (arrows, numbered) with associated Sertoli cells (SC). D: cortical microtubules (red) are visible in the seminiferous tubule epithelium and $\gamma$-tubulin is observed in spermatid centrosomal implantation fossa (green, arrowheads). Inset: TACC3 (green), microtubules (red) and DNA (blue). TACC3 is not present in late-stage spermatid centrioles. SP: spermatocyte cells show abundant cortical microtubules (red) co-stained with $\gamma$-tubulin (green; blue, DNA). E-F: multinuclear late-stage spermatids (DNA, blue) with attached sperm axoneme (short arrows) embedded in seminiferous tubule epithelium (E: DIC). Microtubules (red; DNA, blue) are present in epithelium (red) with weak cytoplasmic $\gamma$-tubulin foci (arrows). The sperm centriolar fossa do not label with $\gamma$-tubulin, as observed in adjacent spermatocyte cells (green, arrows). F: inset, TACC3 (green), microtubules (red) and DNA (blue). No TACC3 is associated with late-stage spermatid nuclei. G-H: multinuclear late-stage spermatid with attached sperm axonemes (G: DIC, short arrows) within seminiferous tubule epithelium. H: $\gamma$-tubulin (green), microtubules (red) and DNA (blue) shows no discernible labeling of spermatid centriolar fossa or assembled microtubules in the epithelium. SP: early-stage spermatocyte (DNA, blue) with a pair of $\gamma$-tubulin foci (green, arrows) within small microtubule asters (red). Inset: TACC3 (green), microtubules (red) and DNA (blue) shows only TACC3 in the SP cytoplasm (arrowheads). All images are $\gamma$-tubulin (green), microtubules (red) and DNA (blue) immunolabeled except B: GFP-CETN2-expressing centrioles (green) and DNA (blue). All insets: TACC3 (green), microtubules (red) and DNA (blue). Scale bars= 5-μm.
